# Comparing different definitions of prediabetes with subsequent risk of diabetes: an individual participant data meta-analysis involving 76 513 individuals and 8208 cases of incident diabetes

**DOI:** 10.1136/bmjdrc-2019-000794

**Published:** 2019-12-29

**Authors:** Crystal Man Ying Lee, Stephen Colagiuri, Mark Woodward, Edward W Gregg, Robert Adams, Fereidoun Azizi, Rafael Gabriel, Tiffany K Gill, Clicerio Gonzalez, Allison Hodge, David R Jacobs Jr, Joshua J Joseph, Davood Khalili, Dianna J Magliano, Kirsten Mehlig, Roger Milne, Gita Mishra, Morgana Mongraw-Chaffin, Julie A Pasco, Masaru Sakurai, Pamela J Schreiner, Elizabeth Selvin, Jonathan E Shaw, Gary Wittert, Hiroshi Yatsuya, Rachel R Huxley

**Affiliations:** 1 School of Psychology and Public Health, La Trobe University, Bundoora, Victoria, Australia; 2 Boden Collaboration for Obesity, Nutrition and Exercise & Eating Disorders, University of Sydney, Sydney, New South Wales, Australia; 3 The George Institute for Global Health, University of New South Wales, Sydney, New South Wales, Australia; 4 The George Institute for Global Health, University of Oxford, Oxford, UK; 5 Department of Epidemiology, Johns Hopkins University, Baltimore, Maryland, USA; 6 Department of Epidemiology and Statistics, School of Public Health, Imperial College London, London, UK; 7 Adelaide Institute for Sleep Health, College of Medicine and Public Health, Flinders University, Adelaide, South Australia, Australia; 8 Respiratory and Sleep Service, Southern Adelaide Local Health Network, SA Health, Adelaide, South Australia, Australia; 9 Faculty of Health and Medical Sciences, Adelaide Medical School, The University of Adelaide, Adelaide, South Australia, Australia; 10 Endocrine Research Center, Research Institute for Endocrine Sciences, Shahid Beheshti University of Medical Sciences, Tehran, Iran; 11 National School of Public Health, National Institute of Health Carlos III, Madrid, Spain; 12 Unidad de Investigación en Diabetes y Riesgo Cardiovascular, Instituto Nacional de Salud Publica, Cuernavaca, Morelos, Mexico; 13 Cancer Epidemiology Centre, Cancer Council Victoria, Melbourne, Victoria, Australia; 14 Centre for Epidemiology and Biostatistics, Melbourne School of Population and Global Health, The University of Melbourne, Melbourne, Victoria, Australia; 15 Division of Epidemiology and Community Health, School of Public Health, University of Minnesota, Minneapolis, Minnesota, USA; 16 Division of Endocrinology, Diabetes and Metabolism, Department of Medicine, Ohio State University Wexner Medical Center, Columbus, Ohio, USA; 17 Prevention of Metabolic Disorders Research Center, Research Institute for Endocrine Sciences, Shahid Beheshti University of Medical Sciences, Tehran, Iran; 18 Department of Biostatistics and Epidemiology, Research Institute for Endocrine Sciences, Shahid Beheshti University of Medical Sciences, Tehran, Iran; 19 Diabetes and Population Health, Baker Heart and Diabetes Institute, Melbourne, Victoria, Australia; 20 Department of Public Health and Community Medicine, Institute of Medicine, University of Gothenburg, Goteborg, Sweden; 21 School of Public Health, Faculty of Medicine, University of Queensland, Brisbane, Queensland, Australia; 22 Department of Epidemiology & Prevention, Wake Forest University School of Medicine, Winston-Salem, North Carolina, USA; 23 Department of Clinical and Biomedical Sciences, Barwon Health, The University of Melbourne, Geelong, Victoria, Australia; 24 School of Medicine, Faculty of Health, Deakin University, Geelong, Victoria, Australia; 25 Department of Epidemiology and Preventive Medicine, Monash University, Melbourne, Victoria, Australia; 26 Department of Social and Environmental Medicine, Kanazawa Medical University, Uchinada, Ishikawa, Japan; 27 Department of Epidemiology, Johns Hopkins Bloomberg School of Public Health, Baltimore, Maryland, USA; 28 Clinical Diabetes and Epidemiology, Baker Heart and Diabetes Institute, Melbourne, Victoria, Australia; 29 Discipline of Medicine, Adelaide Medical School, The University of Adelaide, Adelaide, South Australia, Australia; 30 Department of Public Health, School of Medicine, Fujita Health University, Toyoake, Aichi, Japan; 31 Department of Public Health and Health Systems, Nagoya University Graduate School of Medicine, Nagoya, Aichi, Japan; 32 College of Science, Health and Engineering, La Trobe University, Bundoora, Victoria, Australia

**Keywords:** pre-diabetes, fasting blood glucose, glycated hemoglobin, incidence

## Abstract

**Objective:**

There are currently five widely used definition of prediabetes. We compared the ability of these to predict 5-year conversion to diabetes and investigated whether there were other cut-points identifying risk of progression to diabetes that may be more useful.

**Research design and methods:**

We conducted an individual participant meta-analysis using longitudinal data included in the Obesity, Diabetes and Cardiovascular Disease Collaboration. Cox regression models were used to obtain study-specific HRs for incident diabetes associated with each prediabetes definition. Harrell’s C-statistics were used to estimate how well each prediabetes definition discriminated 5-year risk of diabetes. Spline and receiver operating characteristic curve (ROC) analyses were used to identify alternative cut-points.

**Results:**

Sixteen studies, with 76 513 participants and 8208 incident diabetes cases, were available. Compared with normoglycemia, current prediabetes definitions were associated with four to eight times higher diabetes risk (HRs (95% CIs): 3.78 (3.11 to 4.60) to 8.36 (4.88 to 14.33)) and all definitions discriminated 5-year diabetes risk with good accuracy (C-statistics 0.79–0.81). Cut-points identified through spline analysis were fasting plasma glucose (FPG) 5.1 mmol/L and glycated hemoglobin (HbA1c) 5.0% (31 mmol/mol) and cut-points identified through ROC analysis were FPG 5.6 mmol/L, 2-hour postload glucose 7.0 mmol/L and HbA1c 5.6% (38 mmol/mol).

**Conclusions:**

In terms of identifying individuals at greatest risk of developing diabetes within 5 years, using prediabetes definitions that have lower values produced non-significant gain. Therefore, deciding which definition to use will ultimately depend on the goal for identifying individuals at risk of diabetes.

**Significance of this study**

**What is already known about this subject?**

- Prediabetes comprises heterogeneous states of impaired fasting glucose, impaired glucose tolerance or elevated glycated hemoglobin (HbA1c), which may have different underlying pathophysiologies.
- The definition of prediabetes has changed over time and there is no consensus as to the optimal definition for prediabetes in terms of identifying individuals at greatest risk of progressing to overt diabetes.

**What are the new findings?**

- All five current prediabetes definitions identified individuals at risk of developing diabetes within 5 years with similar accuracy.
- Cut-points identified in this study were lower than the lower thresholds of the current definitions of prediabetes.
- Using diabetes definitions that have lower values to identify individuals at greatest risk of developing diabetes within 5 years did not significantly improve prediction.

**How might these results change the focus of research or clinical practice?**

- Deciding which definition to use in research or clinical practice will depend on the goal for identifying individuals at risk of diabetes.
- Two-hour postload plasma glucose (2hPG) and HbA1c data on the same participants are required to directly compare the strength of association and discriminatory ability of 2hPG and HBA1c-based prediabetes definitions.

## Introduction

Type 2 diabetes mellitus is one of the most important causes of morbidity and mortality globally. There are an estimated 425 million individuals aged 20–79 years worldwide with diabetes, 90% of whom have type 2 diabetes and 212 million of them are living with undiagnosed diabetes.^1^ As type 2 diabetes is largely lifestyle-related and it is possible to delay or prevent onset through appropriate behavior modification, early identification of individuals most at risk of developing type 2 diabetes has become a cornerstone of public health and diabetes prevention policies.^2 3^

Although the relationship between blood glucose and vascular risk has been shown to be positive and increase monotonically from a low threshold,^4 5^ cut-points for fasting plasma glucose (FPG), 2-hour postload plasma glucose (2hPG) following an oral glucose challenge and glycated hemoglobin (HbA1c) are frequently used to diagnose diabetes and initiate treatment. Individuals with levels of glycemia that fall just below the cut-point for diabetes are considered to have ‘prediabetes’, a term that is often used to help identify individuals at risk of converting to overt diabetes, and who therefore may be most receptive to lifestyle interventions that prevent or delay onset. Prediabetes comprises heterogeneous states of impaired fasting glucose (IFG), impaired glucose tolerance (IGT) or elevated HbA1c.^6^ Although each of these conditions may have different underlying pathophysiologies, they have been reported to have similar diabetes progression rates in the range of 35.5–45.5 per 1000 person-years for current definitions of prediabetes based on these measures.^7 8^

The definition of prediabetes has changed over time and there is currently no consensus as to the optimal definition for prediabetes in terms of identifying individuals at greatest risk of progressing to overt diabetes.^9^ Based on the WHO IGT definition, there are an estimated 352 million adults aged 20–79 years worldwide who are considered to have prediabetes,^1^ a figure that would increase if IFG was also included and increase substantially if a lower FPG threshold was to be adopted, as is advocated by the American Diabetes Association (ADA).^10^ Despite the current lack of evidence regarding how best to define prediabetes and little reliable information regarding the risk of progression from prediabetes to diabetes,^11^ both the UK and the USA have issued guidelines recommending screening for prediabetes.^12 13^ There is a need for substantial evidence that could represent multiple geographical areas/populations in order to document the optimal diagnostic criteria.

The aims of the current study are twofold. First, to determine which of the current definitions of prediabetes, advocated by ADA, WHO and the International Expert Committee (IEC), has the highest discriminatory capacity for identifying individuals who convert to diabetes within 5 years from those who remain diabetes free, and to see how their performance varies by age, sex and geographical location. Second, to explore if there are other cut-points that are more useful in identifying risk of progression to diabetes.

## Research design and methods

The Obesity, Diabetes and Cardiovascular Disease Collaboration (ODCDC) is an international data pooling collaboration established to address outstanding issues of epidemiological and clinical importance involving indices of body size, markers of glucose homeostasis and risk of diabetes in diverse populations.^14^ The collaboration encompasses populations from Asia, Australia, Europe, and North America. The original ODCDC database was developed from a cleaned and coded dataset provided by investigators of the Collaborative Study of Obesity and Diabetes in Adults (CODA) after obtaining permission for data use from investigators of each of the prospective studies included in CODA. In 2016, we contacted investigators of existing and newly identified studies to provide data for all available study visits to develop a more comprehensive and informative dataset for the Collaboration.

In this study, participants with self-reported diabetes or newly diagnosed diabetes at baseline (n=8803) or who lacked information on diabetes status at baseline (n=1891) or follow-up (n=21 165) were excluded from analyses. Participants with missing data on age, body mass index (BMI), systolic blood pressure (SBP) and/or smoking status at baseline were also excluded (n=479).

### Definitions of prediabetes and incident diabetes

Based on the current cut-points recommended by WHO,^15 16^ ADA^13^ and IEC,^17^ we included two FPG-based, one 2hPG-based and two HbA1c-based definitions of prediabetes (table 1). The same cut-points were applied to studies that measured plasma glucose or serum glucose. Prediabetes was defined as FPG 6.1–6.9 mmol/L according to the WHO-FPG cut-points, FPG 5.6–6.9 mmol/L according to the ADA-FPG cut-points, 2hPG 7.8–11.0 mmol/L according to both WHO and ADA, HbA1c 5.7%–6.4% (39–47 mmol/mol) according to the ADA-HbA1c cut-points and HbA1c 6.0%–6.4% (42–47 mmol/mol) according to the IEC-HbA1c cut-points. To standardize diabetes definitions across studies and to take into account that the definitions may have changed during the study, incident diabetes identified in this study was based on blood tests, self-report and/or use of antidiabetic medications. Diabetes cases confirmed solely through sources such as registry data, medical records, etc were not considered here. For FPG-related analyses, we used incident diabetes as defined by self-report, use of antidiabetic medications and/or FPG≥7.0 mmol/L at follow-up. For 2hPG-related analyses, incident diabetes was classified by self-report, use of antidiabetic medications, FPG≥7.0 mmol/L and/or 2hPG≥11.1 mmol/L. For HbA1c-related analyses, incident diabetes was identified by self-report, use of antidiabetic medications and/or HbA1c≥6.5% (48 mmol/mol).

**Table 1:** Definitions of glucose tolerance status used in this study

Definitions^13 15–17^	Normal	Prediabetes	Incident diabetes
WHO-FPG	FPG<6.1 mmol/L (110 mg/dL)	FPG 6.1–6.9 mmol/L (110–124 mg/dL)	Self-report, use of antidiabetic medications and/or FPG≥7.0 mmol/L (126 mg/dL) at follow-up.
ADA-FPG	FPG<5.6 mmol/L (101 mg/dL)	FPG 5.6–6.9 mmol/L (101–124 mg/dL)	
2hPG	2hPG<7.8 mmol/L (141 mg/dL)	2hPG 7.8–11.0 mmol/L (141–198 mg/dL)	Self-report, use of antidiabetic medications, FPG≥7.0 mmol/L (126 mg/dL) and/or 2hPG≥11.1 mmol/L (200 mg/dL) at follow-up.
ADA-HbA1c	HbA1c<5.7% (39 mmol/mol)	HbA1c 5.7%–6.4% (39–47 mmol/mol)	Self-report, use of antidiabetic medications and/or HbA1c≥6.5% (48 mmol/mol) at follow-up.
IEC-HbA1c	HbA1c<6.0% (42 mmol/mol)	HbA1c 6.0%–6.4% (42–47 mmol/mol)	

ADA, American Diabetes Association; FPG, fasting plasma glucose; HbA1c, glycated hemoglobin; 2hPG, 2-hour postload plasma glucose; IEC, International Expert Committee.

Time to diabetes was calculated as the time between baseline visit and diagnosis of diabetes based on blood testing during a follow-up visit. For participants who self-reported having diabetes or were on antidiabetic medications at follow-up, time to diabetes was estimated as the half-way point between the visit before diabetes was self-reported and the visit when diabetes was reported. Where age at diagnosis of diabetes was available, time to diabetes was calculated as the difference between age at baseline and age at diagnosis of diabetes. Participants who were lost to follow-up or free from diabetes by the end of the study period were censored. Each individual’s follow-up was censored at the time of death or last contact with the respective cohort study.

### Statistical analysis

Progression rates from prediabetes to diabetes were estimated for each prediabetes definitions. Diabetes progression rate per 1000 person-years was calculated as the number of participants with prediabetes at baseline who were diagnosed with diabetes during follow-up divided by the total follow-up years of all participants with prediabetes at baseline, and multiplied by 1000. Cox proportional hazard regression models were used to obtain study-specific HRs and 95% CIs for incident diabetes that were associated with each of the five definitions. Normoglycemia or the state of non-prediabetes and diabetes (table 1), that is below each distinct index threshold of the respective measure, was the reference. Models were adjusted for age and sex and for age, sex, BMI, SBP, cigarette smoking and, where available, family history of diabetes. Random effects meta-analyses were used to pool study-specific log HRs to obtain overall estimate for each prediabetes definition. I^2^ statistics were used to quantify heterogeneity. The analyses were repeated by sex, age group, BMI-defined obesity status and country/geographical region, and tests for heterogeneity across subgroups were obtained by meta-regression. Sensitivity analysis was conducted by excluding family history of diabetes in the multiple adjusted model. Analyses were repeated including only those studies that had measured both FPG and 2hPG or FPG and HbA1c at baseline to allow direct comparison of estimates between FPG and 2hPG and between FPG and HbA1c. For these analyses, the definition of diabetes was modified to self-report, use of antidiabetic medication, FPG≥7.0 mmol/L and/or 2hPG≥11.1 mmol/L in the FPG/2hPG analyses; and self-report, use of antidiabetic medication, FPG≥7.0 mmol/L and/or HbA1c≥6.5% in the FPG/HbA1c analyses.

Harrell’s C-statistics, stratified by study and adjusted for the above-mentioned covariates, were used to estimate how well each prediabetes definition discriminated between those who developed diabetes over 5 years and those who did not. Participants who did not develop diabetes in the first 5 years from baseline were censored. Random effects meta-analyses were used to obtain pooled C-statistics for each prediabetes definition. These analyses were repeated by subgroups mentioned above and tested for heterogeneity using meta-regression. In addition, the analyses were repeated on studies that measured both FPG and 2hPG or FPG and HbA1c at baseline and the modified definitions of diabetes as stated above were used.

Restricted cubic splines with four knots were used to examine the relationship between each of the three baseline measures of glycemia and incident diabetes. Knots were defined based on Harrell’s recommended percentiles.^18^ Reference was chosen as the mean rounded to the nearest whole number. A series of single knot linear splines were fitted to explore if threshold associations exist between each of the three baseline measures of glycemia and incident diabetes. The optimal cut-point was taken to be the knot that corresponded to the smallest Akaike information criterion value. Spline analyses were adjusted for age, sex and study and repeated with adjustments for age, sex, study, BMI, SBP, cigarette smoking and family history of diabetes. In addition, receiver operating characteristic curve (ROC) analyses were conducted to determine optimal cut-points for discriminating 5-year diabetes risk. The optimal cut-point was taken to be the value where the sum of sensitivity and specificity is at maximum and that sensitivity and specificity are both >50% to protect against unacceptable rates of classification error.

Population attributable fraction of diabetes was calculated for each prediabetes definition using the formula: 100×prevalence×(HR–1)/[100+prevalence×(HR–1)]. Age- and sex-adjusted HRs were used in these calculations.

All statistical analyses were performed using Stata/SE V.14.0 (StataCorp, College Station, Texas, USA).

## Results

Sixteen studies contributed information on 76 513 participants in whom 8208 cases of FPG-defined incident diabetes developed over a mean follow-up of 11.1 years (range 4.9–21.7 years). Of these studies, five were from Australia, four from the USA, two each from Japan and Sweden and one each from Iran, Mexico and Spain. While all studies collected FPG (mean 5.2 mmol/L; range of mean values 4.1–5.5 mmol/L), four studies additionally collected 2hPG (mean 6.0 mmol/L; range 5.5–6.1 mmol/L) and five studies collected HbA1c (mean 5.3% (34 mmol/mol); range 5.1%–5.5% (32–37 mmol/mol)). Baseline characteristics of participants by study are shown in online supplementary sTable 1. In brief, mean (SD) age at baseline was 49.6 (12.7) years, mean BMI was 26.3 (5.0) kg/m^2^, mean SBP was 125.0 (19.1) mm Hg, 51.7% of participants were female, 19.1% were current smokers and 20.8% had a family history of diabetes. Progression from prediabetes to diabetes was 45.7 per 1000 person-years (range 20.0–138.4 per 1000 person-years) for WHO-FPG-defined prediabetes, 23.7 per 1000 person-years (range 11.2–80.8 per 1000 person-years) for ADA-FPG-defined prediabetes, 43.8 per 1000 person-years (range 30.5–62.2 per 1000 person-years) for 2hPG, 45.2 per 1000 person-years (range 31.0–87.7 per 1000 person-years) for ADA-HbA1c and 79.4 per 1000 person-years (range 51.1–154.3 per 1000 person-years) for IEC-HbA1c (online supplementary sTable 2).

10.1136/bmjdrc-2019-000794.supp1 Supplementary data

### Association between prediabetes and risk of diabetes

**Figure 1:**
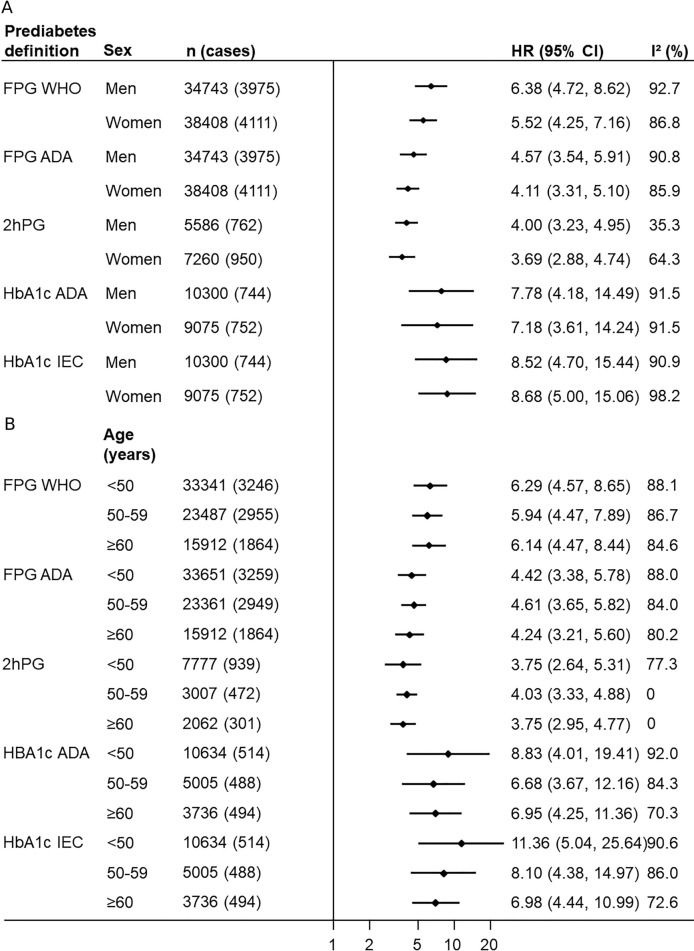
Associations between prediabetes and diabetes by prediabetes definition and (A) sex, (B) age group. HRs adjusted for age, body mass index, systolic blood pressure, smoking and family history of diabetes; normal (ie, non-prediabetes or diabetes) was the reference group. ADA, American Diabetes Association; FPG, fasting plasma glucose; HbA1c, glycated hemoglobin; 2hPG, 2-hour postload plasma glucose; IEC, International Expert Committee. See table 1 for the respective definitions.

From 74 095 participants with measured FPG and no diabetes at baseline, 8208 cases of incident diabetes as defined by self-report, use of antidiabetic medications and/or FPG≥7.0 mmol/L, over 825 051 person-years of follow-up were included in analyses involving FPG. Compared with the normal FPG group, WHO-FPG-defined prediabetes at study baseline was associated with an age- and sex-adjusted HR (95% CIs) of 7.50 (5.86 to 9.60) (online supplementary sFigure 1), which was attenuated with further adjustment (multiple adjusted HR 5.54 (4.31 to 7.12); table 2). When the ADA-FPG cut-points were used, the association between prediabetes and diabetes relative to normal FPG was also strong (multiple adjusted HR 4.17 (3.36 to 5.17); table 2; online supplementary sFigure 2). Heterogeneity was observed between studies for all analyses (I^2^ range 93.3%–94.2%). The results were not significantly different between men and women (p=0.511–0.553), between younger and older age groups (0.873–0.926), nor between countries/geographical regions irrespective of which fasting cut-point was used (0.128–0.133; figure 1; online supplementary sTable 3). The results for WHO-FPG were, however, different between BMI-defined obesity subgroups with stronger associations observed in the normal weight group (multiple adjusted HR: BMI 18.5–24.9 kg/m^2^ 10.49 (7.20 to 15.27); BMI 25.0–29.9 kg/m^2^ 6.56 (4.86 to 8.86); BMI≥30 kg/m^2^ 4.36 (3.36 to 5.68); p<0.001; online supplementary sTable 4). Excluding family history of diabetes from the multiple adjusted model did not materially alter the relationships.

**Table 2:** Pooled HRs for incident diabetes association with prediabetes status at baseline and Harrell’s C-statistics for predicting 5-year risk of diabetes associated with prediabetes status at baseline

Prediabetes definition	Multiple adjusted*				
	N	HR (95% CI)†	I^2^ (%)	C-statistics (95% CI)†	I^2^ (%)
WHO-FPG‡	73 151	5.54 (4.31 to 7.12)	93.9	0.789 (0.772 to 0.807)	63.5
ADA-FPG‡	73 151	4.17 (3.36 to 5.17)	93.3	0.803 (0.787 to 0.819)	62.2
2hPG	12 846	3.78 (3.11 to 4.60)	66.4	0.793 (0.774 to 0.812)	0
ADA-HbA1c	19 375	7.81 (4.32 to 14.14)	94.9	0.811 (0.724 to 0.899)	97.9
IEC-HbA1c	19 375	8.36 (4.88 to 14.33)	93.9	0.802 (0.729 to 0.874)	96.2

*Age, sex, body mass index, systolic blood pressure, smoking and family history of diabetes.

†Normal (non-prediabetes or diabetes) was the reference group, see table 1 for the respective definitions.

‡Family history of diabetes was not adjusted for MESA and Aichi.

ADA, American Diabetes Association; FPG, fasting plasma glucose; HbA1c, glycated hemoglobin; 2hPG, 2-hour postload plasma glucose; IEC, International Expert Committee.

In four studies including 13 536 participants without diabetes (145 206 person-years of follow-up) who had 2hPG measured at baseline, 1787 cases of incident diabetes as defined by self-report, use of antidiabetic medications, FPG≥7.0 mmol/L and/or 2hPG≥11.1 mmol/L were identified. Participants with 2hPG-defined prediabetes had almost four times the risk of diabetes as those with normal 2hPG (multiple adjusted HR: 3.78 (3.11 to 4.60); I^2^=66.4%; table 2; online supplementary sFigure 3). As for FPG-defined prediabetes, there was no evidence of a sex-difference (p=0.702), an age-difference (p=0.929), nor a body size-difference (p=0.052) in the 2hPG association (figure 1; online supplementary sTable 4). One study contributed data to each country/region; there was no observable difference in the associations between the four countries (multiple adjusted HRs range from 3.04 (2.17 to 4.26) for Spain to 4.82 (3.93 to 5.91) for Australia; p=0.303; online supplementary sTable 3). Excluding family history of diabetes in the multiple adjusted model did not materially alter the relationships.

For analyses concerning HbA1c, five studies with 19 975 participants free of diabetes who had HbA1c measured at baseline, 1560 cases of incident diabetes as defined by self-report, use of antidiabetic medications and/or HbA1c≥6.5%, and 145 682 person-years of follow-up were included. Participants with ADA-HbA1c-defined prediabetes had approximately eight times the risk of a subsequent diagnosis of diabetes than those with HbA1c<5.7% (multiple adjusted HR: 7.81 (4.32 to 14.14); I^2^=94.9%; table 2; online supplementary sFigure 4s). Similarly, those with IEC-HbA1c-defined prediabetes had roughly eight times higher risk of developing diabetes than those with normal HbA1c but again with considerable between-study heterogeneity (8.36 (4.88 to 14.33); I^2^=93.9%; table 2; online supplementary sFigure 5). The results were similar between men and women (p=0.921–0.930), between-age groups (0.342–0.655), between countries (0.505–0.555) and between obesity status (0.123–0.145; figure 1; online supplementary sTables 3–4). The results were unaffected by excluding family history of diabetes from the multiple adjusted model.

When the analyses were repeated restricted to studies that had measured both FPG and 2hPG at baseline (n=12 844), the relationship between incident diabetes (defined by self-report, use of antidiabetic medications, FPG≥7.0 mmol/L and/or 2hPG≥11.1 mmol/L) and 2hPG-defined prediabetes had a higher HR than either of the FPG definitions of prediabetes, although CIs overlapped (online supplementary sTable 5). For the analyses on studies that had measured both FPG and HbA1c at baseline (n=16 979), the relationship with incident diabetes (defined by self-report, use of antidiabetic medications, FPG≥7.0 mmol/L and/or HbA1c≥6.5%) was highest for IEC-HbA1c-defined prediabetes, although CIs also overlapped.

### Ability of prediabetes to discriminate between people who developed diabetes over 5 years or not

The discriminatory ability for 5-year risk of diabetes was similar between all five prediabetes definitions (online supplementary sFigures 6–10; table 2). The multiple adjusted C-statistics ranged from 0.789 (0.772 to 0.807) for WHO-FPG-defined prediabetes to 0.811 (0.724 to 0.899) for ADA-HbA1c-defined prediabetes (table 2). Apart from the 2hPG prediabetes definition, between-study heterogeneity was observed in all analyses (I^2^=62.2%–97.9%). The discriminatory ability was not significantly different between men and women (p=0.137–0.938), between-age groups (0.290–0.731), nor between countries/geographical regions (0.401–0.949; online supplementary sTables 6–8). The discriminatory ability for WHO-FPG-defined prediabetes was, however, better in the BMI-defined normal weight group than in groups with higher BMI (multiple adjusted C-statistics: BMI 18.5–24.9 kg/m^2^ 0.795 (0.759 to 0.831); BMI 25.0–29.9 kg/m^2^ 0.770 (0.728 to 0.812); BMI≥30 kg/m^2^ 0.734 (0.698 to 0.771); p=0.003; online supplementary sTable 9).

When the analyses were restricted to those studies that had measured both FPG and 2hPG at baseline, the C-statistic was higher for the 2hPG-defined prediabetes (0.792 (0.773 to 0.811)) when compared with the WHO-FPG-defined prediabetes (0.738 (0.718 to 0.758); online supplementary sTable 5). For analyses that were restricted to studies that had measured both FPG and HbA1c, the C-statistics were not different between the prediabetes definitions.

### Shape of relationships between measures of glycemia and incident diabetes

The relationship between 2hPG and incident diabetes appeared log-linear from 2hPG 5.0 mmol/L to below the cut-point for diabetes (=11.0 mmol/L, online supplementary sFigure 11a). In contrast, a non-linear relationship was observed between FPG and incident diabetes with the angle of the slope increasing at approximately FPG 4.5, 5.0 and 5.5 mmol/L (online supplementary sFigure 11b). A non-linear relationship was similarly observed between HbA1c and incident diabetes with the change in slope occurring at approximately HbA1c 4.5% (26 mmol/mol), 5.0% (31 mmol/mol) and 5.5% (37 mmol/mol; online supplementary sFigure 11c).

Based on multiple adjusted single knot linear spline models, the mathematically optimal cut-points associated with incident diabetes were FPG 5.1 mmol/L and HbA1c 5.0% (31 mmol/mol). A cut-point was not derived for 2hPG due to the log-linear relationship observed. The multiple adjusted C-statistics for prediabetes based on these cut-points were 0.783 (0.762 to 0.804) (I^2^=79.9%) for FPG and 0.754 (0.692 to 0.817) (I^2^=94.7%) for HbA1c. The proportions of participants classified as having prediabetes at baseline according to these cut-points were 56.7% for FPG and 77.8% for HbA1c (table 3). Progression from prediabetes to diabetes based on these cut-points were 14.4 per 1000 person-years for FPG and 13.7 per 1000 person-years for HbA1c.

**Table 3:** Characteristics of currently used, and mathematically derived, prediabetes cut-points for predicting 5-year diabetes risk

	Cut-point	Area under the curve	Sensitivity (%)	Specificity (%)	Positive predicted value (%)	Negative predicted value (%)	Baseline prediabetes prevalence (%)	Age-adjsuted and sex-adjusted HR	Population attributable fraction (%)
FPG (mmol/L)									
Derived from ROC	5.6*	0.706 (0.697 to 0.715)	64.1 (62.3 to 65.9)	77.0 (76.7 to 77.3)	9.8 (9.4 to 10.2)	98.2 (98.1 to 98.3)	24.6	5.24	51.1
WHO-FPG	6.1*	0.665 (0.656 to 0.674)	39.1 (37.3 to 41.0)	93.8 (93.7 to 94.0)	19.9 (18.8 to 20.9)	97.5 (97.4 to 97.6)	7.4	7.50	32.5
ADA-FPG	5.6*	0.706 (0.697 to 0.715)	64.1 (62.3 to 65.9)	77.0 (76.7 to 77.3)	9.8 (9.4 to 10.2)	98.2 (98.1 to 98.3)	24.6	5.24	51.1
Derived from spline	5.1*	0.641 (0.634 to 0.648)	83.9 (82.4 to 85.2)	44.3 (44.0 to 44.7)	5.6 (5.3 to 5.8)	98.6 (98.5 to 98.7)	56.7	3.84	61.7
2hPG (mmol/L)									
Derived from ROC	7.0*	0.715 (0.695 to 0.734)	62.0 (58.1 to 65.9)	80.9 (80.2 to 81.6)	13.5 (12.2 to 14.8)	97.8 (97.5 to 98.1)	21.1	4.00	38.8
WHO/ADA-2hPG	7.8*	0.700 (0.680 to 0.720)	50.2 (46.2 to 54.3)	89.8 (89.2 to 90.3)	19.0 (17.2 to 21.0)	97.4 (97.1 to 97.7)	12.1	4.63	30.5
HbA1c (%)									
Derived from ROC	5.6	0.762 (0.747 to 0.777)	72.8 (69.8 to 75.7)	79.6 (79.1 to 80.2)	14.3 (13.3 to 15.4)	98.4 (98.2 to 98.6)	22.7	10.12	67.4
ADA-HbA1c	5.7	0.748 (0.732 to 0.764)	64.7 (61.5 to 67.9)	85.0 (84.4 to 85.5)	16.7 (15.5 to 18.0)	98.1 (97.9 to 98.3)	17.3	9.76	60.2
IEC-HbA1c	6.0	0.679 (0.663 to 0.695)	40.4 (37.2 to 43.8)	95.4 (95.0 to 95.7)	28.9 (26.4 to 31.5)	97.2 (96.9 to 97.4)	6.2	10.72	37.6
Derived from spline	5.0	0.590 (0.582 to 0.598)	94.9 (93.3 to 96.3)	23.0 (22.4 to 23.6)	5.44 (5.09 to 5.81)	99.0 (98.6 to 99.3)	77.8	4.75	74.5

*To convert glucose from mmol/L to mg/dL, divide value by 0.0555.

ADA, American Diabetes Association; FPG, fasting plasma glucose; HbA1c, glycated hemoglobin; 2hPG, 2-hour postload plasma glucose; IEC, International Expert Committee.

According to the ROC analysis, the optimal cut-points for discriminating 5-year risk of diabetes were FPG 5.6 mmol/L (sensitivity=64.1%, specificity=77.0%), 2hPG 7.0 mmol/L (sensitivity=62.0%, specificity=80.9%) and HbA1c 5.6% (38 mmol/mol; sensitivity=72.8%, specificity=79.6%). Compared with these cut-points, current prediabetes definitions with higher cut-points (ie, WHO-FPG, 2hPG and IEC-HbA1c) have significantly lower sensitivity but higher specificity and positive predictive values. In contrast, cut-points derived from spline analysis have substantially higher sensitivity but lower specificity and positive predictive values (table 3).

The fraction of diabetes attributable to prediabetes ranged from 32.5% to 61.7% for FPG, 30.5% to 38.8% for 2hPG and 37.6% to 74.5% for HbA1c (table 3).

## Conclusions

Prediabetes is a contentious term for levels of glycemia that are elevated above the normal range but below the level used to define diabetes, as many people classified with prediabetes do not progress to overt diabetes and may even revert to normoglycemia.^11 19^ Adding to the complexity is that, depending on region of the world, up to five different methods for defining prediabetes are in use. The results of our individual participant meta-analysis showed that all current prediabetes definitions were associated with an increased risk of diagnosed diabetes and all of them identified people at high risk for a subsequent diabetes diagnosis within 5 years with reasonably good accuracy. All variables in the multiple-adjusted models were associated with diabetes risk and the association was strongest for family history of diabetes (HRs 1.53–1.64). Our findings were consistent across groups that differed on the basis of sex, age and geographical region. Furthermore, cut-points identified here were lower than those used in current prediabetes definitions.

Similar to our study, a recent systematic review reported the risk of developing diabetes varied by prediabetes definitions and found no clear pattern of difference between geographical regions.^11^ The review also showed that for people with prediabetes, the overall risk of diabetes increased over time while the likelihood of regression to normoglycemia decreased over time. Surprisingly though, regression to normoglycemia was observed even after 11 years of follow-up.

A US study reported small, but significant, differences in C-statistics for predicting diagnosed diabetes between the prediabetes definitions analyzed here, with higher values observed in FPG-based than HbA1c-based definitions.^20^ In contrast, C-statistics in our study were higher for HbA1c-based than FPG-based definitions in the subgroup with both FPG and HbA1c data, although CIs overlapped. Similarly, higher C-statistics, with overlapping CIs, were observed for the 2hPG definition when compared with FPG-based definitions. Nevertheless, the difference between the highest and lowest C-statistics was small, hence, impact on the overall discriminatory accuracy between the definitions in practice would be negligible.

In addition to comparing the relationship between current prediabetes definitions and incident diabetes, we identified mathematically optimal definitions of prediabetes for FPG and HbA1c. As reported in a Dutch study, non-linear relationships were observed for FPG and HbA1c and incident diabetes.^21^ Our results showed the slope was steeper from approximately 5.0 mmol/L for FPG and from approximately 5.0% (31 mmol/mol) for HbA1c. Similarly, an Israeli study found men with higher FPG within the normal range had progressively increased risk of diabetes compared with men with FPG<4.5 mmol/L.^22^ Furthermore, our results from single knot spline analysis suggested that the optimal cut-points for FPG or HbA1c associated with incident diabetes fell within the normoglycemic range while cut-points identified through ROC analysis were in line with the ADA definitions possibly because similar approach was used (ie, sensitivity and specificity had to be ≥50%). Other studies have also reported optimal cut-points that are below the lower limit of current prediabetes definitions.^23 24^ Whether a lower cut-point is considered appropriate or not will depend on the goal (eg, risk stratification and prediction or targeting intervention strategies and prevention). Lowering the current prediabetes cut-points to the level suggested here would identify substantially more participants with prediabetes at baseline but the rate of progression from prediabetes to diabetes would be lower. As there is limited evidence of benefit of intervention in individuals with IFG,^25 26^ let alone normoglycemia, it seems inappropriate to recommend lowering of cut-points for prediabetes for the purpose of initiating treatment such as pharmacotherapy. Moreover, reclassifying otherwise healthy individuals as having prediabetes may confer psychological distress or economic harm.^27 28^ On the other hand, lowering the cut-points may offer more opportunity to prevent progression to diabetes through lifestyle interventions at a population level.

The strength of our study was the use of individual participant data from 16 studies. Nevertheless, a few limitations warrant mention. Between-study heterogeneity was generally high across all analyses; analysis by subgroups did not identify possible sources of heterogeneity. Variability of HbA1c assays used between studies and within studies over time may have partly contributed to the high heterogeneity in analyses related to HbA1c. Nevertheless, four of the five studies that measured HbA1c conducted baseline and last follow-up visits over similar periods. We were unable to directly compare the strength of association and discriminatory ability of 2hPG-based and HbA1c-based prediabetes definitions in the same participants as only one study had all three measures of glycemia. Furthermore, those who died before follow-up were more likely to have developed diabetes which would have been missed by our studies, given the increased risk of death associated with diabetes.^20 29–32^ If mortality differed between the prediabetes categories, this might lead to different degrees of underestimation of true incidence between the categories. The lack of studies that had collected 2hPG and/or HbA1c also reduced the generalizability of the results by country/geographical region as most of the subgroups included only one study. Moreover, we were unable to examine how the relationship may vary by ethnic/race group. Furthermore, unlike mortality as an outcome which is accompanied by a date of death, time to diabetes is an approximation.

All current prediabetes definitions were associated with greater risk of diabetes relative to people with lower glucose concentrations and identified people at risk of developing diabetes within 5 years with similar accuracy. Therefore, deciding which definition to use will ultimately depend on the allocation of healthcare resources available to intervene in individuals designated at high risk, and the need to balance sensitivity with specificity. Unsurprisingly, using a lower glycemic threshold to define prediabetes will increase the number of individuals who may qualify for an intervention but at the risk of treating many individuals who are less likely to progress to diabetes. As suggested in the WHO/International Diabetes Federation 2006 report,^15^ including known diabetes risk factors in the assessment of risk rather than basing it on a single measure of glycemia would be a better and more nuanced approach to identifying those most at risk of developing diabetes and in need of subsequent intervention.

## References

[1] International Diabetes Federation IDF diabetes atlas. 18th edn Brussels: International Diabetes Federation, 2017.

[2] Australian Government Department of Health Australian National diabetes strategy 2016-2020. Canberra: Commonwealth of Australia, 2015.

[3] National Institute for Health and Care Excellence Type 2 diabetes prevention: population and community-level interventions. Public health guideline [PH35]. Available: https://www.nice.org.uk/guidance/ph35 [Accessed 18 Dec 2018].

[4] Levitan EB, Song Y, Ford ES, et al Is nondiabetic hyperglycemia a risk factor for cardiovascular disease? A meta-analysis of prospective studies. Arch Intern Med 2004;164:2147–55. 10.1001/archinte.164.19.2147 15505129

[5] Lawes CMM, Parag V, Bennett DA, et al Blood glucose and risk of cardiovascular disease in the Asia Pacific region. Diabetes Care 2004;27:2836–42. 10.2337/diacare.27.12.2836 15562194

[6] Tabak AG, Herder C, Rathmann W, et al Prediabetes: a high-risk state for developing diabetes. Lancet 2012;379:2279–90. 22683128 10.1016/S0140-6736(12)60283-9 PMC3891203

[7] Faerch K, Borch-Johnsen K, Holst JJ, et al Pathophysiology and aetiology of impaired fasting glycaemia and impaired glucose tolerance: does it matter for prevention and treatment of type 2 diabetes? Diabetologia 2009;52:1714–23. 10.1007/s00125-009-1443-3 19590846

[8] Morris DH, Khunti K, Achana F, et al Progression rates from HbA1c 6.0-6.4% and other prediabetes definitions to type 2 diabetes: a meta-analysis. Diabetologia 2013;56:1489–93. 10.1007/s00125-013-2902-4 23584433

[9] Lee CM, Colagiuri S Diagnostic criteria and classification : Bonora E, DeFronzo R, Diabetes. epidemiology, genetics, pathogenesis, diagnosis, prevention, and treatment (PP. 23-39. Cham: Springer International Publishing, 2018.

[10] Borch-Johnsen K, Colagiuri S, Balkau B, et al Creating a pandemic of prediabetes: the proposed new diagnostic criteria for impaired fasting glycaemia. Diabetologia 2004;47:1396–402. 10.1007/s00125-004-1468-6 15278279

[11] Richter B, Hemmingsen B, Metzendorf M-I, et al Development of type 2 diabetes mellitus in people with intermediate hyperglycaemia. Cochrane Database Syst Rev 2018;10 10.1002/14651858.CD012661.pub2 PMC6516891 30371961

[12] National Institute for Health and Care Excellence Type 2 diabetes: prevention in people at high risk, 2017 Available: https://www.nice.org.uk/guidance/ph38 [Accessed 11 Dec 2018].

[13] American Diabetes Association 2. Classification and Diagnosis of Diabetes: Standards of Medical Care in Diabetes-2018. Diabetes Care 2018;41:S13–27. 10.2337/dc18-S002 29222373

[14] Lee CMY, Woodward M, Pandeya N, et al Comparison of relationships between four common anthropometric measures and incident diabetes. Diabetes Res Clin Pract 2017;132:36–44. 10.1016/j.diabres.2017.07.022 28783531 PMC5728360

[15] World Health Organization,, International Diabetes Federation Definition and diagnosis of diabetes mellitus and intermediate hyperglycemia: report of a WHO/IDF consultation. Geneva: World Health Organization, 2006.

[16] World Health Organization Use of glycated haemoglobin (HbA1c) in the diagnosis of diabetes mellitus. Abbreviated report of a who consultation. Geneva: World Health Organization, 2011. 26158184

[17] The International Expert Committee. International Expert Committee report on the role of the A1c assay in the diagnosis of diabetes. Diabetes Care 2009;32:1327–34. 19502545 10.2337/dc09-9033 PMC2699715

[18] Harrell FE Regression modelling strategies: with application to linear models, logistic regression, and survival analysis. New York: Springer, 2001.

[19] de Abreu L, Holloway KL, Kotowicz MA, et al Dysglycaemia and other predictors for progression or regression from impaired fasting glucose to diabetes or normoglycaemia. J Diabetes Res 2015;2015:1–8. 10.1155/2015/373762 PMC4530268 26273669

[20] Warren B, Pankow JS, Matsushita K, et al Comparative prognostic performance of definitions of prediabetes: a prospective cohort analysis of the Atherosclerosis risk in communities (ARIC) study. Lancet Diabetes Endocrinol 2017;5:34–42. 10.1016/S2213-8587(16)30321-7 27863979 PMC5183486

[21] Ruijgrok C, Dekker JM, Beulens JW, et al Size and shape of the associations of glucose, HbA_1c_, insulin and HOMA-IR with incident type 2 diabetes: the Hoorn Study. Diabetologia 2018;61:93–100. 10.1007/s00125-017-4452-7 29018885 PMC6448924

[22] Tirosh A, Shai I, Tekes-Manova D, et al Normal fasting plasma glucose levels and type 2 diabetes in young men. N Engl J Med 2005;353:1454–62. 10.1056/NEJMoa050080 16207847

[23] Mukai N, Doi Y, Ninomiya T, et al Cut-Off values of fasting and post-load plasma glucose and HbA1c for predicting type 2 diabetes in community-dwelling Japanese subjects: the Hisayama study. Diabet Med 2012;29:99–106. 10.1111/j.1464-5491.2011.03378.x 21726278

[24] Ryu S, Shin H, Chang Y, et al Should the lower limit of impaired fasting glucose be reduced from 110 mg/dL in Korea? Metabolism 2006;55:489–93. 10.1016/j.metabol.2005.10.010 16546479

[25] Saito T, Watanabe M, Nishida J, et al Lifestyle modification and prevention of type 2 diabetes in overweight Japanese with impaired fasting glucose levels: a randomized controlled trial. Arch Intern Med 2011;171:1352–60. 10.1001/archinternmed.2011.275 21824948

[26] le Roux CW, Astrup A, Fujioka K, et al 3 years of liraglutide versus placebo for type 2 diabetes risk reduction and weight management in individuals with prediabetes: a randomised, double-blind trial. Lancet 2017;389:1399–409. 10.1016/S0140-6736(17)30069-7 28237263

[27] Yudkin JS, Montori VM The epidemic of pre-diabetes: the medicine and the politics. BMJ 2014;349:g4485 10.1136/bmj.g4485 25028385 PMC4707710

[28] Vathesatogkit P, Sritara P, Kimman M, et al Associations of lifestyle factors, disease history and awareness with health-related quality of life in a Thai population. PLoS One 2012;7:e49921 10.1371/journal.pone.0049921 23189172 PMC3506606

[29] DECODE Study Group, the European Diabetes Epidemiology Group Glucose tolerance and cardiovascular mortality: comparison of fasting and 2-hour diagnostic criteria. Arch Intern Med 2001;161:397–405. 10.1001/archinte.161.3.397 11176766

[30] Barr ELM, Zimmet PZ, Welborn TA, et al Risk of cardiovascular and all-cause mortality in individuals with diabetes mellitus, impaired fasting glucose, and impaired glucose tolerance: the Australian diabetes, obesity, and lifestyle study (AusDiab). Circulation 2007;116:151–7. 10.1161/CIRCULATIONAHA.106.685628 17576864

[31] Hirakawa Y, Ninomiya T, Mukai N, et al Association between glucose tolerance level and cancer death in a general Japanese population: the Hisayama study. Am J Epidemiol 2012;176:856–64. 10.1093/aje/kws178 23100249

[32] Vistisen D, Witte DR, Brunner EJ, et al Risk of cardiovascular disease and death in individuals with prediabetes defined by different criteria: the Whitehall II study. Diabetes Care 2018;41:899–906. 10.2337/dc17-2530 29453200 PMC6463620

