# Supplementary material for: Comparing different definitions of prediabetes with subsequent risk of diabetes: an individual participant data meta-analysis involving 76 513 individuals and 8208 cases of incident diabetes
Source: BMJ Open Diabetes Res Care. 2019 Dec 29;7(1):e000794. doi: 10.1136/bmjdrc-2019-000794 (PMC6936411; doi:10.1136/bmjdrc-2019-000794)
Supplement: Supplementary data [file bmjdrc-2019-000794supp001.pdf]

Online supplementary material

Comparing different definitions of pre-diabetes with subsequent risk of diabetes: An individual participant data meta-analysis involving 76,513 individuals and 8208 cases of incident diabetes

Obesity, Diabetes and Cardiovascular Disease Collaboration

Study investigators

We would like to thank investigators of the following studies for contributing data to this project: Aichi Workers' Cohort Study (Hiroshi Yatsuya); Atherosclerosis Risk in Communities Study (June Stevens, Elizabeth Selvin); Australian Diabetes, Obesity and Lifestyle Study (Jonathan Shaw, Dianna Magliano); Coronary Artery Risk Development in Young Adults Study (Pamela Schreiner, David Jacobs Jr); Florey Adelaide Male Ageing Study (Gary Wittert, Sean Martin); Geelong Osteoporosis Study (Julie Pasco); Jackson Heart Study (Morgana Mongraw-Chaffin, Alain Bertoni, Joshua Joseph); Melbourne Collaborative Cohort Study (Graham Giles, Allison Hodge, Roger Milne); Mexico City Diabetes Study (Clicerio Gonzalez); Multi-Ethnic Study of Atherosclerosis (Morgana Mongraw-Chaffin, Alain Bertoni); North West Adelaide Health Study (Kay Price, Robert Adams, Tiffany Gill); Prospective Population Study of Women in Gothenburg (Lauren Lissner, Kirsten Mehlig); Tehran Lipid and Glucose Study (Fereidoun Azizi, Davood Khalili); Toyama Prefecture Study (Masaru Sakurai); Uppsala Longitudinal Study of Adult Men (Bjorn Zethelius, Vilmantas Giedraitis); Variability of Insulin with Visceral Adiposity project (Rafael Gabriel);

sTable 1: Characteristics of participants by study: mean (standard deviation) unless stated

Study	Country	Baseline year	N	Female (%)	Follow-up (years)	DM incidence cases*	Baseline							
							Age (years)	FPG† (mmol/L)	2hPG† (mmol/L)	HbA1c (%)	BMI (kg/m^2)	SBP (mmHg)	Current smoker (%)	Family diabetes history (%)
**AICHI (Ref1)**	Japan	2005, 2007	5281	19.3	5.4 (2.0)	259	47.4 (7.0)	5.1 (0.5)	-	-	22.9 (2.8)	124.3 (16.0)	24.7	-
**ARIC (Ref2)**	USA	1987-1989	12061	54.5	13.2 (8.1)	2286	54.0 (5.7)	5.5 (0.5)	-	-	27.2 (5.0)	119.7 (18.0)	25.6	22.3
**AUSDIAB (Ref3)**	Australia	1999-2000	6558	55.0	10.1 (3.2)	464	50.5 (12.7)	5.4 (0.6)	6.0 (1.8)	5.1 (0.3)	26.7 (4.7)	127.8 (17.4)	12.1	18.3
**CARDIA (Ref4)**	USA	1985-1986	4797	55.8	21.4 (6.0)	626	24.9 (3.6)	4.5 (0.5)	-	-	24.5 (5.0)	110.4 (11.1)	29.1	15.5
**FAMAS (Ref5)**	Australia	2002-2005	798	0	4.9 (0.6)	59	53.7 (11.1)	4.7 (0.7)	-	5.5 (0.4)	28.3 (4.1)	136.4 (18.1)	17.8	27.8
**GOS (Ref6)**	Australia	1994-1997	639	100	9.5 (1.2)	30	47.3 (14.3)	5.3 (0.5)	-	-	26.2 (5.1)	120.2 (19.8)	14.4	36.0
**JHS (Ref7)**	USA	2000-2004	3419	63.4	7.3 (1.8)	465	53.6 (12.5)	5.0 (0.5)	-	5.5 (0.5)	31.4 (7.2)	125.9 (16.1)	11.8	45.5
**MCCS (Ref8)**	Australia	1990-1994	17025	61.2	11.7 (1.8)	1445	54.8 (8.4)	5.5 (0.5)	-	-	26.5 (4.2)	135.3 (18.1)	8.3	17.6
**MCDS (Ref9)**	Mexico	1990-1992	1690	59.3	11.8 (5.8)	333	46.3 (8.0)	4.6 (0.7)	5.9 (2.0)	-	28.1 (4.3)	116.6 (16.7)	32.8	32.5

**MESA (Ref10)**	USA	2000-2002	5532	53.2	7.7 (2.7)	564	51.6 (10.2)	5.0 (0.6)	-	-	28.0 (5.3)	125.1 (20.9)	12.8	-
**NWAHS (Ref11)**	Australia	1999-2000, 2002-2003	3062	53.2	6.7 (2.3)	155	50.4 (15.6)	5.0 (0.7)	-	5.4 (0.5)	27.7 (5.2)	127.4 (17.8)	20.2	10.3
**PSWG (Ref12)**	Sweden	1968	1320	100	18.3 (7.2)	113	46.7 (6.2)	4.1 (0.7)	-	-	24.0 (3.7)	132.5 (21.3)	41.8	15.2
**TLGS (Ref13)**	Iran	1999-2001	4409	57.4	11.1 (3.7)	780	41.1 (13.9)	5.0 (0.6)	6.1 (2.0)	-	26.6 (4.6)	118.0 (17.9)	13.6	25.8
**TOYAMA (Ref14)**	Japan	2009	6507	33.6	5.0 (1.4)	185	41.5 (11.8)	5.2 (0.6)	-	5.3 (0.4)	22.9 (3.3)	116.8 (16.3)	27.9	15.4
**ULSAM (Ref15)**	Sweden	1970-1973	1881	0	21.7 (10.0)	294	49.6 (06)	4.9 (0.5)	-	-	25.0 (3.1)	132.5 (17.6)	34.4	15.5
**VIVA (Ref16)**	Spain	1996-1997	1534	56.1	11.0 (1.7)	150	48.3 (8.4)	5.1 (0.6)	5.5 (1.7)	-	27.5 (4.4)	125.2 (19.5)	30.3	21.9
**Total**			76513	51.7	11.1 (6.3)	8208	49.6 (12.7)	5.2 (0.6)	6.0 (1.9)	5.3 (0.4)	26.3 (5.0)	125.0 (19.1)	19.1	20.8

*Diabetes is defined as FPG ≥ 7.0 mmol/L, self-report, or use of anti-diabetic medication;

† To convert from mmol/L to mg/dl: divide value by 0.0555

DM = diabetes mellitus; WHO = World Health Organization; FPG = fasting plasma glucose; ADA = American Diabetes Association; 2hPG = 2 hour post-load plasma glucose; HbA1c = glycated haemoglobin; BMI = body mass index; SBP = systolic blood pressure; AICHI = Aichi Workers' Cohort Study; ARIC = Atherosclerosis Risk in Communities Study; AUSDIAB = Australian Diabetes, Obesity and Lifestyle Study; CARDIA = Coronary Artery Risk Development in Young Adults Study; FAMAS = Florey Adelaide Male Ageing Study; GOS = Geelong Osteoporosis Study; JHS = Jackson Heart Study; MCCS = Melbourne Collaborative Cohort Study; MCDS = Mexico City Diabetes Study; MESA = Multi-Ethnic Study of Atherosclerosis; NWAHS = North

West Adelaide Health Study; PSWG = Prospective Population Study of Women in Gothenburg; TLGS = Tehran Lipid and Glucose Study; TOYAMA = Toyama Prefecture Study; ULSAM = Uppsala Longitudinal Study of Adult Men; VIVA = Variability of Insulin with Visceral Adiposity project;

Ref1 Yatsuya H, Li Y, Hirakawa Y, Ota A et al. A point system for predicting 10-year risk of developing type 2 diabetes mellitus in Japanese men: Aichi Workers' Cohort Study. J Epidemiol 2018;28:347-52.

Ref2 The ARIC investigators. The Atherosclerosis Risk in Communities (ARIC) Study: design and objectives. Am J Epidemiol 1989;129:687-702.

Ref3 Dunstan DW, Zimmet PZ, Welborn TA et al. The Australian Diabetes, Obesity and Lifestyle Study (AusDiab) – methods and response rates. Diabetes Res Clin Pract 2002;57:119-29.

Ref4 Friedman GD, Cutter GR, Donahue RP et al. CARDIA: Study design, recruitment and some characteristics of the examined subjects. J Clin Epidemiol 1988;41:1105-16.

Ref5 Martin S, Haren M, Taylor A et al. Cohort profile: the Florey Adelaide Male Ageing Study (FAMAS). Int J Epidemiol 2007;36:302-6.

Ref6 Pasco JA, Nicholson GC, Kotowicz MA. Cohort profile: Geelong Osteoporosis Study. Int J Epidemiol 2012;41:1565-75.

Ref7 Sempos CT, Bild DE, Manolio TA. Overview of the Jackson Heart Study: a study of cardiovascular diseases in African American men and women. Am J Med Sci 1999;317:142-6.

Ref8 Milne RL, Fletcher AS, MacInnis RJ et al. Cohort profile: The Melbourne Collaborative Cohort Study (Health 2020). Int J Epidemiol 2017;46:1757-1757i.

Ref9 Gonzalez C, Stern MP, Gonzalez E et al. The Mexico City Diabetes Study: a population-based approach to the study of genetic and environmental interactions in the pathogenesis of obesity and diabetes. Nutr Rev 1999;57:S71-6.

Ref10 Bild DE, Bluemke DA, Burke GL et al. Multi-Ethnic Study of Atherosclerosis: objectives and design. Am J Epidemiol 2002;156:871-81.

Ref11 Grant JF, Taylor AW, Ruffin RE et al. Cohort profile: The North West Adelaide Health Study 9NWAHS). Int J Epidemiol 2009;38:1479-86.

Ref12 Bengtsson C, Ahlqwist M, Andersson K et al. The prospective Population Study of Women in Gothenburg, Sweden, 1968-69 to 1992-93. A 24-year follow-up stud with special reference to participation, representativeness, and mortality. Scand J Prim Health Care 1997;15:214-9.

Ref13 Hadaegh F, Harati H, Ghasemi A et al. Using common clinical data improves the prediction of abnormal glucose tolerance by the new criteria of impaired fasting glucose: Tehran Lipid and Glucose study. Diabetes Res Clin Pract 2007;77:459-64.

Ref14 Sakurai M, Nakamura K, Miura K et al. Dietary carbohydrate intake, presence of obesity and the incident risk of type 2 diabetes in Japanese men. J Diabetes Investig 2016;7:343-51.

Ref15 Lithell H, Sundstrom J, Arnlov J et al. Epidemiological and clinical studies on insulin resistance and diabetes. Ups J Med Sci 2000;105:135-50.

Ref16 Pladevall M, Singal B, Williams LK et al. A single factor underlies the metabolic syndrome. A confirmatory factor analysis. Diabetes Care 2006;29:113-22.

sTable 2: Prediabetes conversion to diabetes by pre-diabetes definition

Study	Pre-diabetes conversion to diabetes (per 1000 person years)				
	WHO-FPG	ADA-FPG	2hPG	ADA-HbA1c	IEC-HbA1c
AICHI	87.3	36.3	-	-	-
ARIC	57.5	29.0	-	-	-
AUSDIAB	36.2	15.2	30.5	87.7	154.3
CARDIA	20.0	15.5	-	-	-
FAMAS	113.0	80.8	-	48.8	95.6
GOS	22.4	11.2	-	-	-
JHS	136.8	76.6	-	49.0	83.4
MCCS	24.6	13.5	-	-	-
MCDS	92.0	69.7	54.1	-	-
MESA	138.4	70.3	-	-	-
NWAHS	53.3	26.3	-	31.0	51.1
PSWG	49.3	18.6	-	-	-
TLGS	107.8	66.4	62.2	-	-
TOYAMA	99.3	36.6	-	42.0	113.4
ULSAM	32.2	17.9	-	-	-
VIVA	30.8	21.0	36.5	-	-
**Total**	45.7	23.7	43.8	45.2	79.4

WHO = World Health Organization; FPG = fasting plasma glucose; ADA = American Diabetes Association; 2hPG = 2 hour post-load plasma glucose; HbA1c = glycated haemoglobin; IEC = International Expert Committee; AICHI = Aichi Workers' Cohort Study; ARIC = Atherosclerosis Risk in Communities Study; AUSDIAB = Australian Diabetes, Obesity and Lifestyle Study; CARDIA = Coronary Artery Risk Development in Young Adults Study; FAMAS = Florey Adelaide Male Ageing Study; GOS = Geelong Osteoporosis Study; JHS = Jackson Heart Study; MCCS = Melbourne Collaborative Cohort Study; MCDS = Mexico City Diabetes Study; MESA = Multi-Ethnic Study of Atherosclerosis; NWAHS = North West Adelaide Health Study; PSWG = Prospective Population Study of Women in Gothenburg; TLGS = Tehran Lipid and Glucose Study; TOYAMA = Toyama Prefecture Study; ULSAM = Uppsala Longitudinal Study of Adult Men; VIVA = Variability of Insulin with Visceral Adiposity project;

sTable 3: Pooled hazard ratios (HR) and 95% confidence intervals (CI) for incident diabetes associated with pre-diabetes status at baseline by country/geographical region

Pre-diabetes definition	Subgroup	Multiple adjusted*			Heterogeneity between subgroup p-value
		n	Hazard ratio (95% CI)†	I^2 (%)	
**WHO-FPG**	**Australia**	27368	4.67 (3.04, 7.19)	89.8	0.128
	**Europe**	4683	3.59 (2.67, 4.82)	8.8	
	**Iran**	4289	5.41 (4.45, 6.58)	-	
	**Japan**	9739	12.21 (7.19, 20.74)	84.2	
	**North America**	27072	5.92 (3.71, 9.44)	95.5	
**ADA-FPG**	**Australia**	27368	3.56 (2.73, 4.64)	75.1	0.133
	**Europe**	4683	2.70 (2.20, 3.32)	0	
	**Iran**	4289	4.98 (4.27, 5.80)	-	
	**Japan**	9739	7.32 (3.88, 13.80)	88.0	
	**North America**	27072	4.57 (2.89, 7.24)	96.7	
**2hPG**	**Australia**	5763	4.82 (3.93, 5.91)	-	0.303
	**Iran**	3956	3.98 (3.38, 4.68)	-	
	**Mexico**	1644	3.20 (2.52, 4.06)	-	
	**Spain**	1483	3.04 (2.17, 4.26)	-	
**ADA-HbA1c**	**Australia**	9720	5.68 (2.77, 11.64)	93.5	0.505
	**Japan**	6481	29.95 (19.09, 46.99)	-	
	**USA**	3174	5.67 (4.58, 7.02)	-	
**IEC-HbA1c**	**Australia**	9720	6.54 (3.70, 11.57)	87.0	0.555
	**Japan**	6481	23.35 (16.51, 33.03)	-	
	**USA**	3174	6.26 (5.20, 7.53)	-	

WHO = World Health Organization; FPG = fasting plasma glucose; ADA = American Diabetes Association; 2hPG = 2 hour post-load plasma glucose; HbA1c = glycated haemoglobin; IEC = International Expert Committee; Europe = Spain, Sweden; North America = Mexico, USA; CI = confidence intervals;

* Adjusted for age, body mass index, systolic blood pressure, smoking, and family history of diabetes

† Normal (i.e. non pre-diabetes or diabetes) was the reference group, see Table 1 for the respective definitions;

Lee CMY, *et al. BMJ Open Diab Res Care* 2019; 7:e000794. doi: 10.1136/bmjdrc-2019-000794

sTable 4: Pooled hazard ratios (HR) and 95% confidence intervals (CI) for incident diabetes associated with pre-diabetes status at baseline by obesity status

Pre-diabetes definition	Subgroup	Multiple adjusted*			Heterogeneity between subgroup p-value
		n	Hazard ratio (95% CI)†	I^2 (%)	
**WHO-FPG**	**BMI 18.5-24.9**	29924	10.49 (7.2, 15.27)	83.3	<0.001
	**BMI 25.0-29.9**	26314	6.56 (4.86, 8.86)	79.0	
	**BMI ≥30.0**	14025	4.36 (3.36, 5.68)	75.2	
**ADA-FPG**	**BMI 18.5-24.9**	29924	5.92 (4.23, 8.28)	85.0	0.049
	**BMI 25.0-29.9**	26314	4.71 (3.68, 6.04)	86.6	
	**BMI ≥30.0**	14025	3.51 (2.74, 4.49)	86.9	
**2hPG**	**BMI 18.5-24.9**	4485	5.71 (3.40, 9.60)	60.6	0.052
	**BMI 25.0-29.9**	5410	4.02 (3.13, 5.17)	51.7	
	**BMI ≥30.0**	2806	3.36 (2.62, 4.32)	55.4	
**ADA-HbA1c**	**BMI 18.5-24.9**	8433	13.36 (3.35, 53.35)	92.8	0.145
	**BMI 25.0-29.9**	6265	8.46 (4.07, 17.59)	90.7	
	**BMI ≥30.0**	4013	5.05 (4.23, 6.01)	0	
**IEC-HbA1c**	**BMI 18.5-24.9**	8433	12.51 (3.50, 44.74)	92.1	0.123
	**BMI 25.0-29.9**	6265	10.44 (4.78, 22.80)	92.2	
	**BMI ≥30.0**	4013	5.41 (4.55, 6.43)	0	

WHO = World Health Organization; FPG = fasting plasma glucose; ADA = American Diabetes Association; 2hPG = 2 hour post-load plasma glucose; HbA1c = glycated haemoglobin; IEC = International Expert Committee; Europe = Spain, Sweden; North America = Mexico, USA; CI = confidence intervals;

* Adjusted for age, sex, systolic blood pressure, smoking, and family history of diabetes

† Normal (i.e. non pre-diabetes or diabetes) was the reference group, see Table 1 for the respective definitions;

sTable 5: Pooled hazard ratios (HR) for incident diabetes†‡ associated with pre-diabetes status at baseline and Harrell's c-statistics for predicting 5 year risk of diabetes for studies which measured both FPG and 2hPG or FPG and HbA1c, respectively

Pre-diabetes definition	Multiple adjusted*			
	**Hazard ratio (95% CI)**	**I^2 (%)**	**c-statistics (95%CI)**	**I^2 (%)**
**Based on studied that measured FPG and 2hPG (n=12,844)**				
**WHO-FPG†**	3.61 (2.63, 4.97)	49.2	0.738 (0.718, 0.758)	0
**ADA-FPG†**	3.11 (2.35, 4.11)	83.1	0.757 (0.738, 0.776)	0
**2hPG†**	3.79 (3.12, 4.60)	66.8	0.792 (0.773, 0.811)	0
**Based on studies that measured FPG and HbA1c (n=16,979)**				
**WHO-FPG‡**	5.81 (4.18, 8.06)	81.4	0.766 (0.719, 0.813)	87.3
**ADA-FPG‡**	3.96 (2.82, 5.55)	87.8	0.770 (0.729, 0.812)	85.3
**ADA-HbA1c‡**	6.18 (4.16, 9.18)	89.4	0.785 (0.754, 0.816)	73.7
**IEC-HbA1c‡**	7.13 (4.77, 10.66)	88.2	0.774 (0.738, 0.811)	80.0

*Age, sex, body mass index (BMI), systolic blood pressure, smoking, and family history of diabetes;

† Normal (i.e. non pre-diabetes or diabetes) was the reference group, incident diabetes was self-report, use of anti-diabetic medication, FPG ≥7.0 mmol/L (126 mg/dl), and/or 2hPG ≥11.1 mmol/L (200 mg/dl);

‡ Normal (I.e. non-pre-diabetes or diabetes) was the reference group, incident diabetes was self-report, use of anti-diabetic medication, FPG ≥7.0 mmol/L (126 mg/dl), and/or HbA1c ≥6.5% (48 mmol/mol);

WHO = World Health Organization; FPG = fasting plasma glucose; ADA = American Diabetes Association; 2hPG = 2 hour post-load plasma glucose; HbA1c = glycated haemoglobin; IEC = International Expert Committee;

Lee CMY, *et al. BMJ Open Diab Res Care* 2019; 7:e000794. doi: 10.1136/bmjdrc-2019-000794

sTable 6: Harrell's c-statistics for predicting 5 year risk of diabetes by sex

Pre-diabetes definition	Subgroup	Age adjusted			Multiple adjusted†			Heterogeneity between subgroup p-value
		n	c-statistics (95% CI)*	I^2 (%)	n	c-statistics (95% CI)*	I^2 (%)	
**WHO-FPG**	**Men**	35217	0.714 (0.680, 0.748)	76.7	34743	0.796 (0.780, 0.813)	30.7	0.354
	**Women**	38878	0.674 (0.643, 0.706)	65.2	38408	0.783 (0.762, 0.804)	49.9	
**ADA-FPG**	**Men**	35217	0.753 (0.724, 0.782)	75.7	34743	0.811 (0.792, 0.831)	57.5	0.465
	**Women**	38878	0.730 (0.700, 0.759)	69.0	38408	0.801 (0.781, 0.820)	45.7	
**2hPG**	**Men**	5930	0.771 (0.733, 0.809)	33.5	5586	0.819 (0.786, 0.853)	40.8	0.137
	**Women**	7606	0.724 (0.655, 0.793)	77.5	7260	0.784 (0.758, 0.809)	0	
**ADA-HbA1c**	**Men**	10592	0.768 (0.682, 0.855)	94.6	10300	0.818 (0.738, 0.898)	94.8	0.938
	**Women**	9383	0.741 90.629, 0.853)	95.7	9075	0.822 (.706, 0.938)	98.2	
**IEC-HbA1c**	**Men**	10592	0.739 (0.674, 0.8040	87.0	10300	0.809 (0.753, 0.864)	87.0	0.761
	**Women**	9383	0.715 (0.594, 0.836)	95.3	9075	0.823 (0.707, 0.938)	97.9	

WHO = World Health Organization; FPG = fasting plasma glucose; ADA = American Diabetes Association; 2hPG = 2 hour post-load plasma glucose; HbA1c = glycated haemoglobin; IEC = International Expert Committee; CI = confidence intervals;

* Normal (i.e. non pre-diabetes or diabetes) was the reference group, see Table 1 for the respective definitions;

† Adjusted for age, body mass index, systolic blood pressure, smoking, and family history of diabetes

Lee CMY, *et al. BMJ Open Diab Res Care* 2019; 7:e000794. doi: 10.1136/bmjdrc-2019-000794

sTable 7: Harrell's c-statistics for predicting 5 year risk of diabetes by age

**Pre-diabetes definition**	**Subgroup**	**Sex adjusted**			**Multiple adjusted†**			**Heterogeneity between subgroup p-value**
		**n**	**c-statistics (95% CI)***	**I^2 (%)**	**n**	**c-statistics (95% CI)***	**I^2 (%)**	
**WHO-FPG**	**Age <50 years**	34014	0.619 (0.560, 0.678)	95.0	33646	0.800 (0.783, 0.817)	17.0	0.731
	**Age 50-59 years**	23755	0.673 (0.632, 0.715)	80.9	23487	0.809 (0.736, 0.882)	98.3	
	**Age ≥60 years**	16225	0.680 (0.635, 0.726)	75.0	15912	0.790 (0.759, 0.821)	58.0	
**ADA-FPG**	**Age <50 years**	34014	0.699 (0.661, 0.737)	75.1	33651	0.819 (0.795, 0.842)	54.6	0.551
	**Age 50-59 years**	23755	0.742 (0.713, 0.772)	71.6	23487	0.833 (0.768, 0.899)	98.1	
	**Age ≥60 years**	16291	0.685 (0.605, 0.766)	96.8	15978	0.801 (0.770, 0.832)	66.9	
**2hPG**	**Age <50 years**	8075	0.711 (0.665, 0.757)	47.6	7777	0.798 (0.771, 0.825)	0	0.290
	**Age 50-59 years**	3172	0.710 (0.668, 0.751)	0	3007	0.793 (0.758, 0.827)	4.9	
	**Age ≥60 years**	2289	0.734 (0.674, 0.794)	38.8	2062	0.857 (0.727, 0.986)	96.0	
**ADA-HbA1c**	**Age <50 years**	10866	0.470 (0.644, 0.836)	91.4	10634	0.827 (0.722, 0.932)	95.7	0.398
	**Age 50-59 years**	5168	0.720 (0.637, 0.803)	90.8	5005	0.795 (0.741, 0.849)	79.2	
	**Age ≥60 years**	3941	0.732 90.686, 0.778)	68.3	3736	0.793 (0.745, 0.841)	68.6	
**IEC-HbA1c**	**Age <50 years**	10866	0.664 (0.616, 0.713)	57.7	10634	0.805 (0.714, 0.897)	92.4	0.342
	**Age 50-59 years**	5168	0.699 (0.608, 0.791)	89.9	5005	0.794 (0.769, 0.819)	2.1	
	**Age ≥60 years**	3941	0.693 (0.614, 0.772)	84.6	3736	0.779 (0.749, 0.809)	14.4	

WHO = World Health Organization; FPG = fasting plasma glucose; ADA = American Diabetes Association; 2hPG = 2 hour post-load plasma glucose; HbA1c = glycated haemoglobin; IEC = International Expert Committee; CI = confidence intervals;

* Normal (i.e. non pre-diabetes or diabetes) was the reference group, see Table 1 for the respective definitions;

† Adjusted for sex, body mass index, systolic blood pressure, smoking, and family history of diabetes

Lee CMY, *et al. BMJ Open Diab Res Care* 2019; 7:e000794. doi: 10.1136/bmjdrc-2019-000794

sTable 8: Harrell's c-statistics for predicting 5 year risk of diabetes by country/geographical region

Pre-diabetes definition	Subgroup	Age and sex adjusted			Multiple adjusted†			Heterogeneity between subgroup p-value
		n	c-statistics (95% CI)*	I^2 (%)	n	c-statistics (95% CI)*	I^2 (%)	
**WHO-FPG**	**Australia**	27920	0.701 (0.655, 0.747)	51.9	27368	0.786 (0.762, 0.809)	0	0.949
	**Europe**	4735	0.639 (0.561, 0.717)	0	4683	0.801 (0.721, 0.880)	42.7	
	**Iran**	4407	0.739 (0.712, 0.766)	-	4289	0.763 (0.736, 0.789)	-	
	**Japan**	9739	0.782 (0.734, 0.830)	78.7	9739	0.820 (0.761, 0.879)	85.4	
	**North America**	27294	0.662 (0.624, 0.701)	78.7	27072	0.782 (0.755, 0.810)	74.6	
**ADA-FPG**	**Australia**	27920	0.724 (0.692, 0.756)	26.4	27368	0.786 (0.762, 0.809)	0	0.709
	**Europe**	4735	0.752 (0.684, 0.821)	1.9	4683	0.845 (0.792, 0.899)	0	
	**Iran**	4407	0.776 (0.750, 0.803)	-	4289	0.797 (0.771, 0.822)	-	
	**Japan**	9739	0.799 (0.752, 0.846)	75.4	9739	0.825 (0.771, 0.879)	84.7	
	**North America**	27294	0.708 (0.661, 0.754)	88.5	27072	0.798 (0.777, 0.819)	61.7	
**2hPG**	**Australia**	6297	0.725(0.686, 0.764)	-	5763	0.788 (0.755, 0.821)	-	0.906
	**Iran**	4061	0.776 (0.749, 0.804)	-	3956	0.797 (0.769, 0.825)	-	
	**Mexico**	1644	0.687 (0.629, 0.746)	-	1644	0.790 (0.747, 0.833)	-	
	**Spain**	1534	0.702 (0.565, 0.839)	-	1483	0.800 (0.697, 0.904)	-	
**ADA-HbA1c**	**Australia**	10281	0.709 (0.685, 0.733)	0	9720	0.783 (0.761, 0.805)	0	0.529
	**Japan**	6481	0.905 (0.879, 0.931)	-	6481	0.935 (0.920, 0.950)	-	
	**USA**	3213	0.730 (0.701, 0.759)	-	3174	0.770 (0.744, 0.796)	-	
**IEC-HbA1c**	**Australia**	10281	0.660 (0.587, 0.734)	85.7	9720	0.770 (0.730, 0.810)	66.1	0.401
	**Japan**	6481	0.855 (0.822, 0.887)	-	6481	0.911 (0.891, 0.932)	-	

**USA**	3213	0.727 (0.692, 0.762)	-	3174	0.782 (0.753, 0.811)	-

WHO = World Health Organization; FPG = fasting plasma glucose; ADA = American Diabetes Association; 2hPG = 2 hour post-load plasma glucose; HbA1c = glycated haemoglobin; IEC = International Expert Committee; Europe = Spain, Sweden; North America = Mexico, USA; CI = confidence intervals;

* Normal (i.e. non pre-diabetes or diabetes) was the reference group, see Table 1 for the respective definitions;

† Adjusted for age, sex, body mass index, systolic blood pressure, smoking, and family history of diabetes

sTable 9: Harrell's c-statistics for predicting 5 year risk of diabetes by body mass index (BMI) defined obesity status

Pre-diabetes definition	Subgroup	Age and sex adjusted			Multiple adjusted†			Heterogeneity between subgroup p-value
		n	c-statistics (95% CI)*	I^2 (%)	n	c-statistics (95% CI)*	I^2 (%)	
**WHO-FPG**	**BMI 18.5-24.9**	30247	0.749 (0.710, 0.788)	57.5	29924	0.795 (0.759, 0.831)	56.5	0.033
	**BMI 25.0-29.9**	26709	0.713 (0.695, 0.730)	0	26314	0.770 (0.728, 0.812)	85.6	
	**BMI ≥30.0**	14236	0.681 (0.655, 0.707)	43.9	14025	0.734 (0.698, 0.771)	77.1	
**ADA-FPG**	**BMI 18.5-24.9**	30247	0.774 (0.736, 0.811)	59.8	29924	0.808 (0.775, 0.841)	54.2	0.058
	**BMI 25.0-29.9**	26709	0.739 (0.724, 0.754)	0	26314	0.795 (0.761, 0.828)	80.0	
	**BMI ≥30.0**	14236	0.702 (0.671, 0.734)	65.2	14025	0.755 (0.701, 0.809)	93.1	
**2hPG**	**BMI 18.5-24.9**	4735	0.823 90.650, 0.997)	97.1	4485	0.785 (0.737, 0.833)	0	0.123
	**BMI 25.0-29.9**	5693	0.746 (0.713, 0.778)	0	5410	0.785 (0.755, 0.816)	0	
	**BMI ≥30.0**	2957	0.721 (0.688, 0.753)	0	2806	0.747 (0.705, 0.789)	38.4	
**ADA-HbA1c**	**BMI 18.5-24.9**	8648	0.767 (0.592, 0.942)	95.5	8433	0.804 (0.664, 0.944)	93.5	0.076
	**BMI 25.0-29.9**	6510	0.745 (0.701, 0.788)	45.1	6265	0.778 (0.747, 0.809)	5.9	
	**BMI ≥30.0**	4150	0.708 (0.678, 0.739)	32.0	4013	0.735 (0.712, 0.757)	0	
**IEC-HbA1c**	**BMI 18.5-24.9**	8648	0.748 (0.570, 0.926)	95.5	8433	0.790 (0.659, 0.922)	92.0	0.092
	**BMI 25.0-29.9**	6510	0.713 90.640, 0.787)	79.2	6265	0.765 (0.722, 0.807)	43.4	
	**BMI ≥30.0**	4150	0.677 (0.635, 0.719)	52.0	4013	0.720 (0.676, 0.764)	61.5	

WHO = World Health Organization; FPG = fasting plasma glucose; ADA = American Diabetes Association; 2hPG = 2 hour post-load plasma glucose; HbA1c = glycated haemoglobin; IEC = International Expert Committee; CI = confidence intervals;

* Normal (i.e. non pre-diabetes or diabetes) was the reference group, see Table 1 for the respective definitions;

† Adjusted for age, sex, systolic blood pressure, smoking, and family history of diabetes

sFigure 1: Age- and sex-adjusted pooled hazard ratios (HR) for incident diabetes association with World Health Organization-fasting plasma glucose defined pre-diabetes status (fasting plasma glucose 6.1–6.9 mmol/L (110–124 mg/dl) at baseline

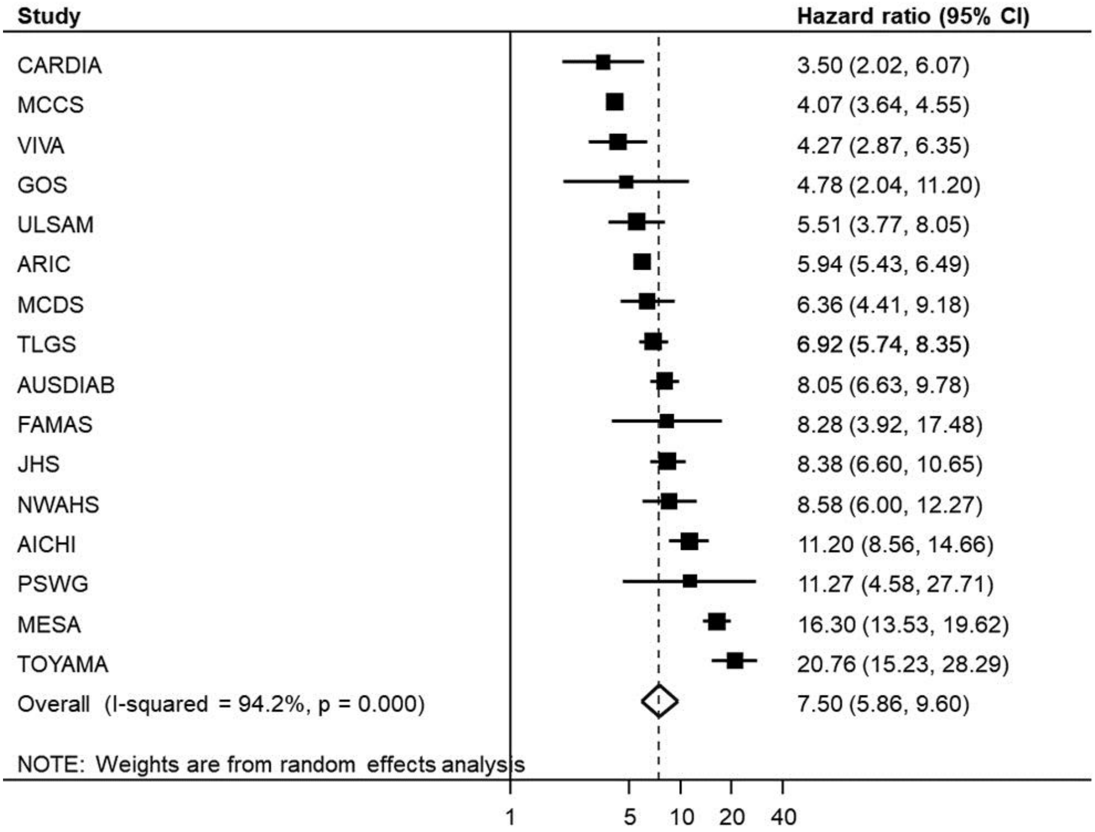

Normal fasting plasma gluocse (fasting plasma glucose <6.1 mmol/L (110 mg/dl)) was the reference group;

sFigure 2: Age- and sex-adjusted pooled hazard ratios (HR) for incident diabetes association with American Diabetes Association-fasting plasma glucose defined pre-diabetes status (fasting plasma glucose 5.6–6.9 mmol/L (101–124 mg/dl) at baseline

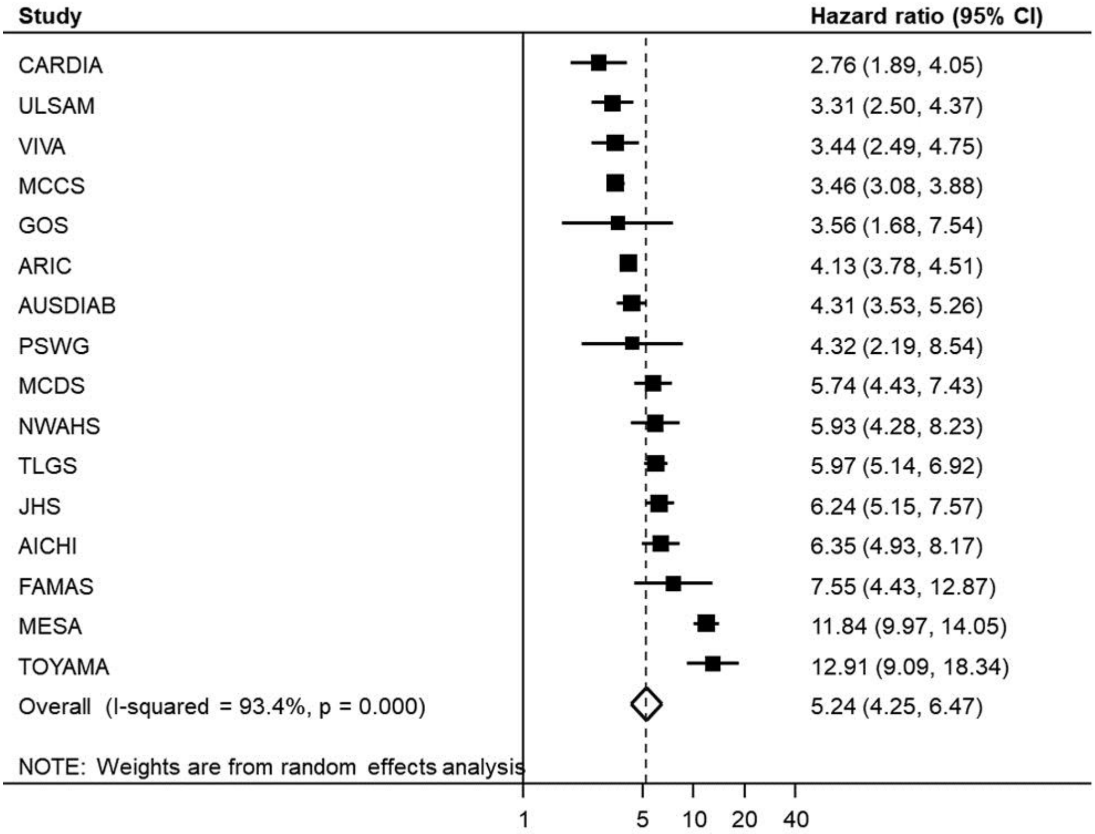

Normal fasting plasma glucose (fasting plasma glucose <5.6 mmol/L (101 mg/dl)) was the reference group;

sFigure 3: Age- and sex-adjusted pooled hazard ratios (HR) for incident diabetes association with 2 hour postload plasma glucose defined pre-diabetes status (2 hour plasma glucose 7.8–11.0 mmol/L (141–198 mg/dl) at baseline

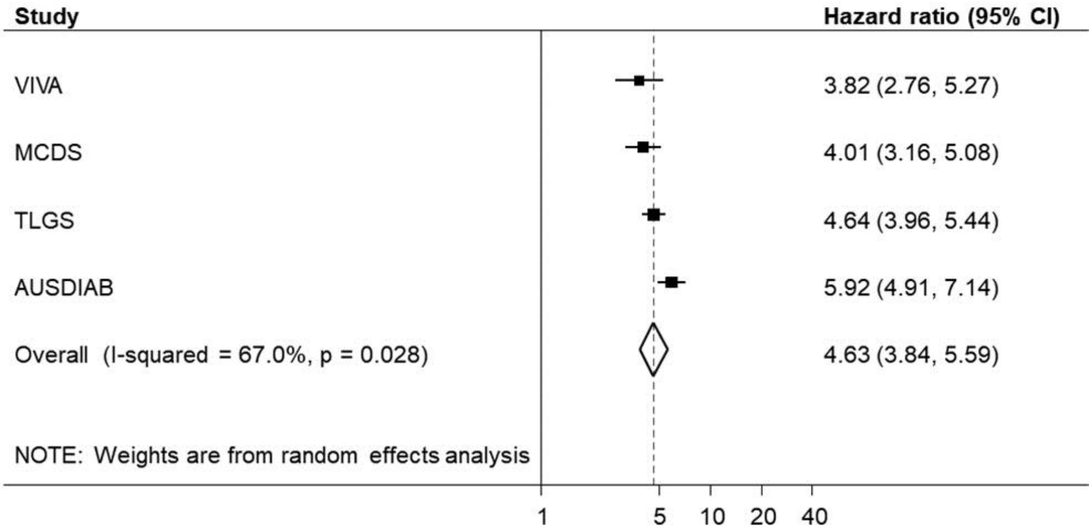

Normal 2 hour plasma glucose (2 hour plasma glucose <7.8 mmol/L (141 mg/dl)) was the reference group;

sFigure 4: Age- and sex-adjusted pooled hazard ratios (HR) for incident diabetes association with American Diabetes Association-HbA1c defined pre-diabetes status (HbA1c 5.7–6.4% (39–47 mmol/mol)) at baseline

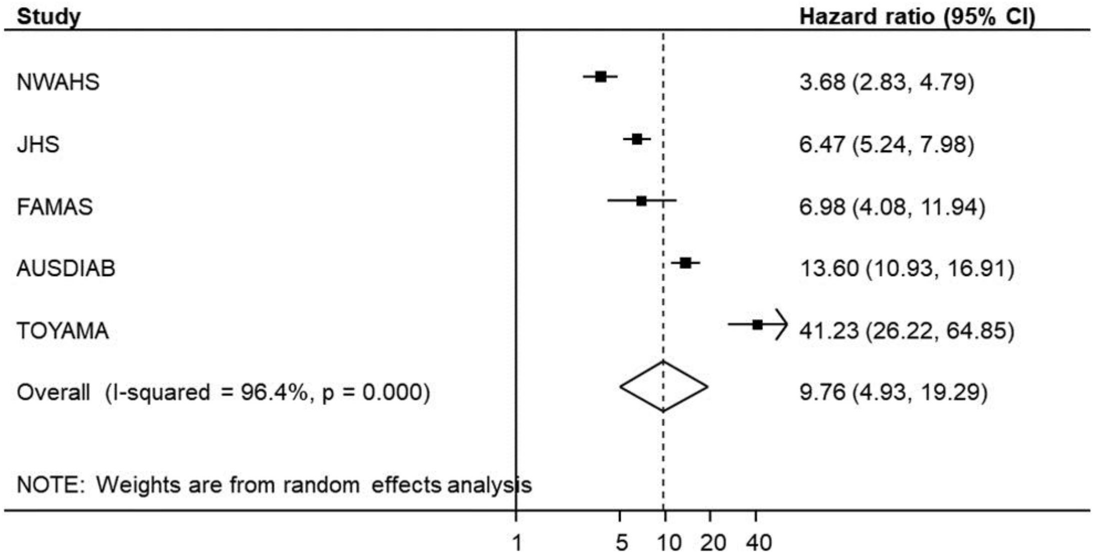

Normal HbA1c (HbA1c <5.7% (39 mmol/mol)) was the reference group;

sFigure 5: Age- and sex-adjusted pooled hazard ratios (HR) for incident diabetes association with International Expert Committee-HbA1c defined pre-diabetes status (HbA1c 6.0–6.4% (42–47 mmol/mol)) at baseline

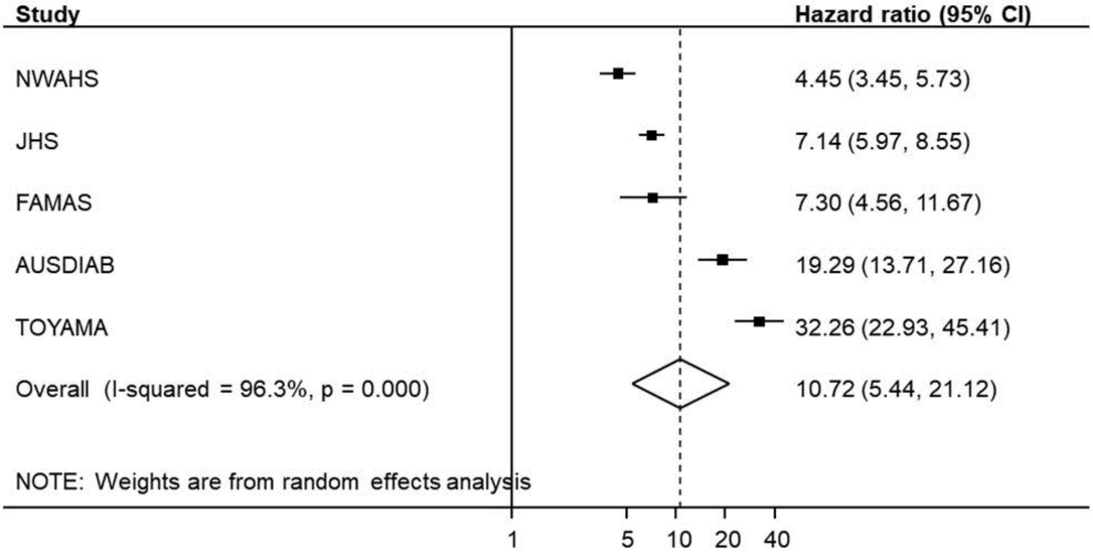

Normal HbA1c (HbA1c<6.0% (42 mmol/mol)) was the reference group;

sFigure 6: Age- and sex-adjusted Harrell's c-statistics for predicting 5 year risk of diabetes associated with World Health Organization-fasting plasma glucose defined pre-diabetes status (fasting plasma glucose 6.1–6.9 mmol/L (110–124 mg/dl) at baseline

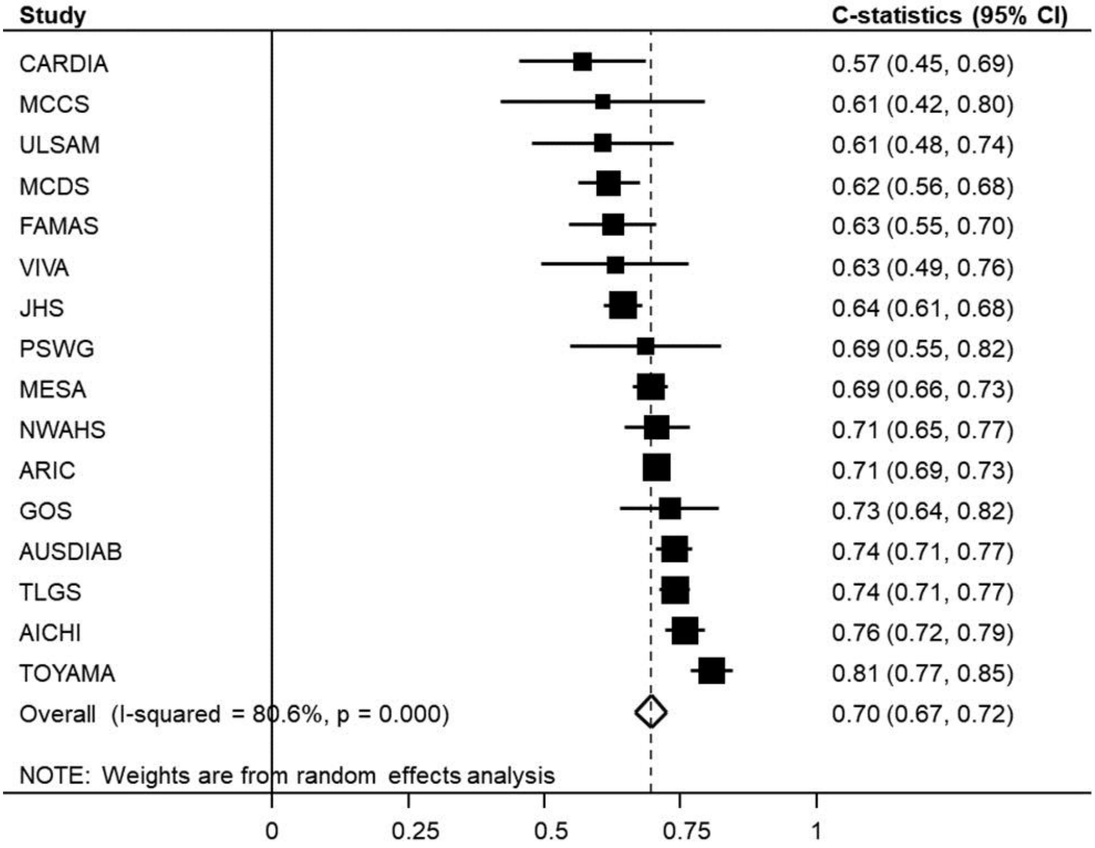

Normal fasting glucose (fasting plasma glucose <6.1 mmol/L (110 mg/dl)) was the reference group;

sFigure 7: Age- and sex-adjusted Harrell's c-statistics for predicting 5 year risk of diabetes associated with American Diabetes Association-fasting plasma glucose defined pre-diabetes status (fasting plasma glucose 5.6–6.9 mmol/L (101–124 mg/dl) at baseline

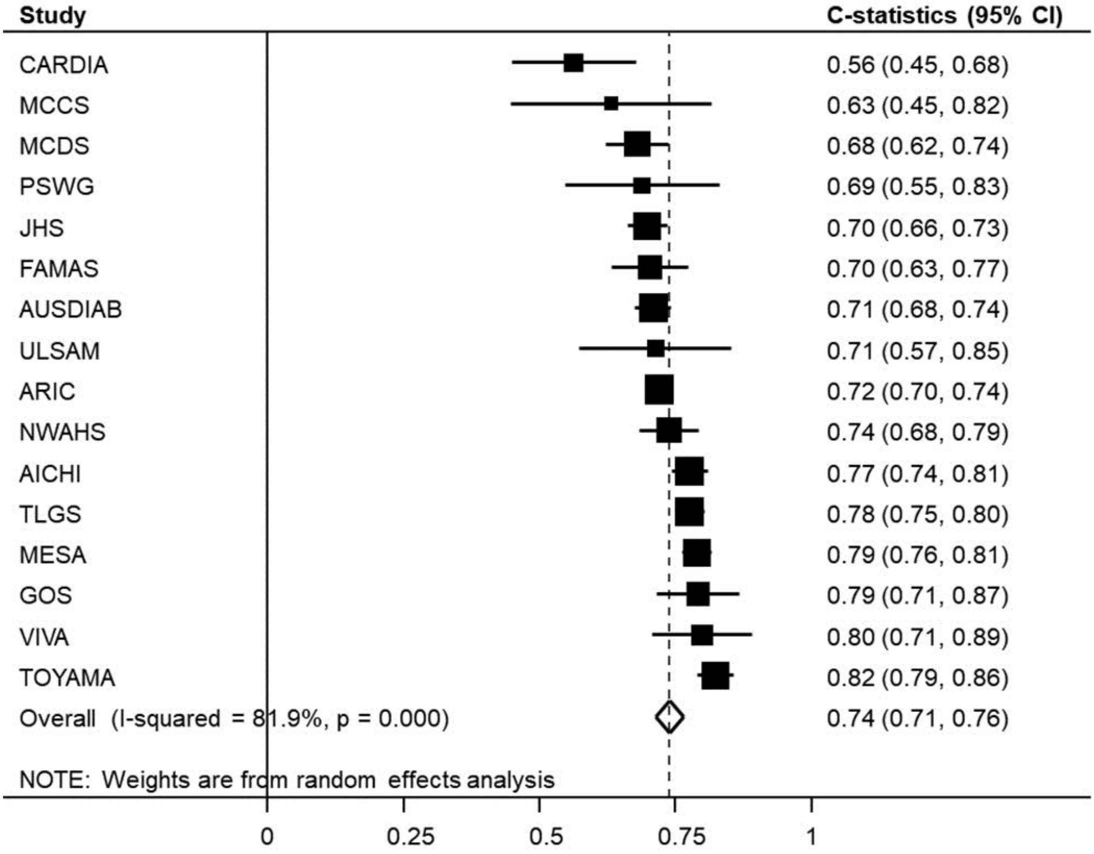

Normal fasting plasma glucose (fasting plama glucose <5.6 mmol/L (101 mg/dl)) was the reference group;

Lee CMY, *et al. BMJ Open Diab Res Care* 2019; 7:e000794. doi: 10.1136/bmjdrc-2019-000794

sFigure 8: Age- and sex-adjusted Harrell's c-statistics for predicting 5 year risk of diabetes associated with 2 hour postload plasma glucose defined pre-diabetes status (2 hour plasma glucose 7.8–11.0 mmol/L (141–198 mg/dl) at baseline

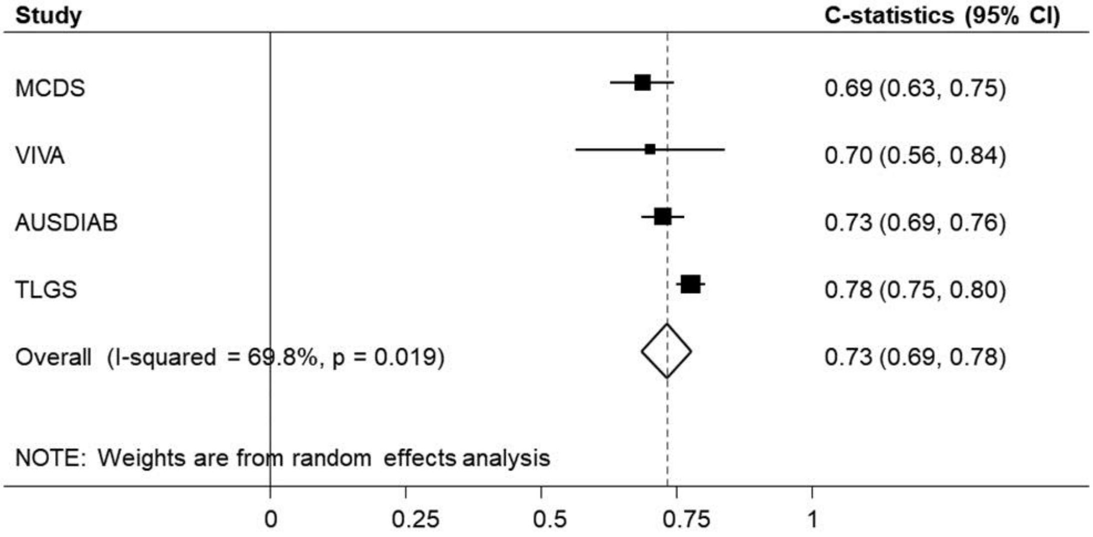

Normal 2 hour plasma glucose (2 hour plasma glucose <7.8 mmol/L (141 mg/dl)) was the reference group;

Lee CMY, *et al. BMJ Open Diab Res Care* 2019; 7:e000794. doi: 10.1136/bmjdrc-2019-000794

sFigure 9: Age- and sex-adjusted Harrell's c-statistics for predicting 5 year risk of diabetes associated with American Diabetes Association-HbA1c defined pre-diabetes status (HbA1c 5.7–6.4% (39–47 mmol/mol))at baseline

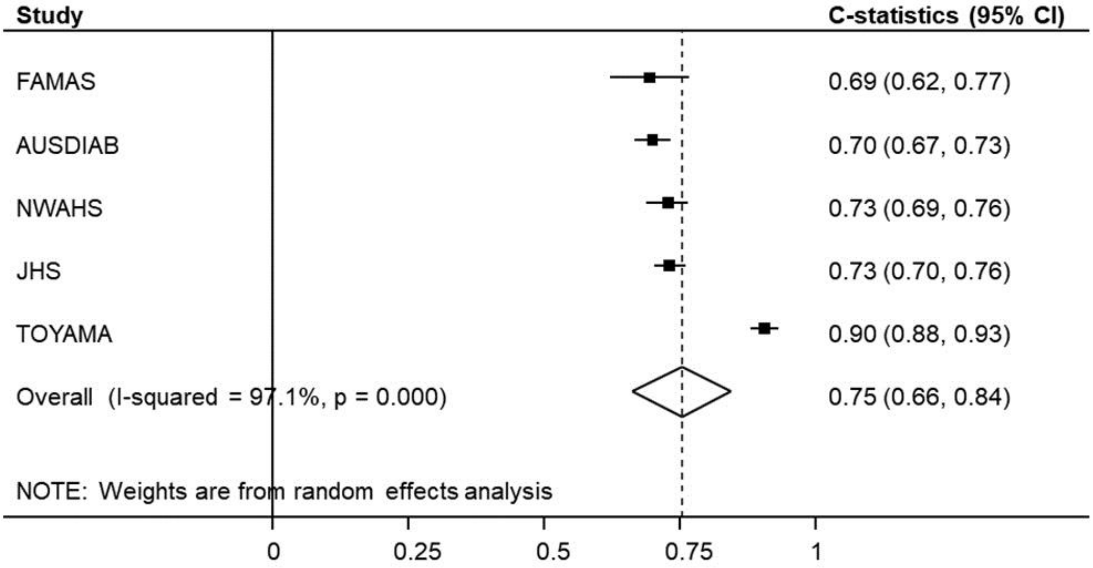

Normal HbA1c (HbA1c <5.7% (39 mmol/mol)) was the reference group;

sFigure 10: Age- and sex-adjusted Harrell's c-statistics for predicting 5 year risk of diabetes associated with International Expert Committee-HbA1c defined pre-diabetes status ((HbA1c 6.0–6.4% (42–47 mmol/mol)) at baseline

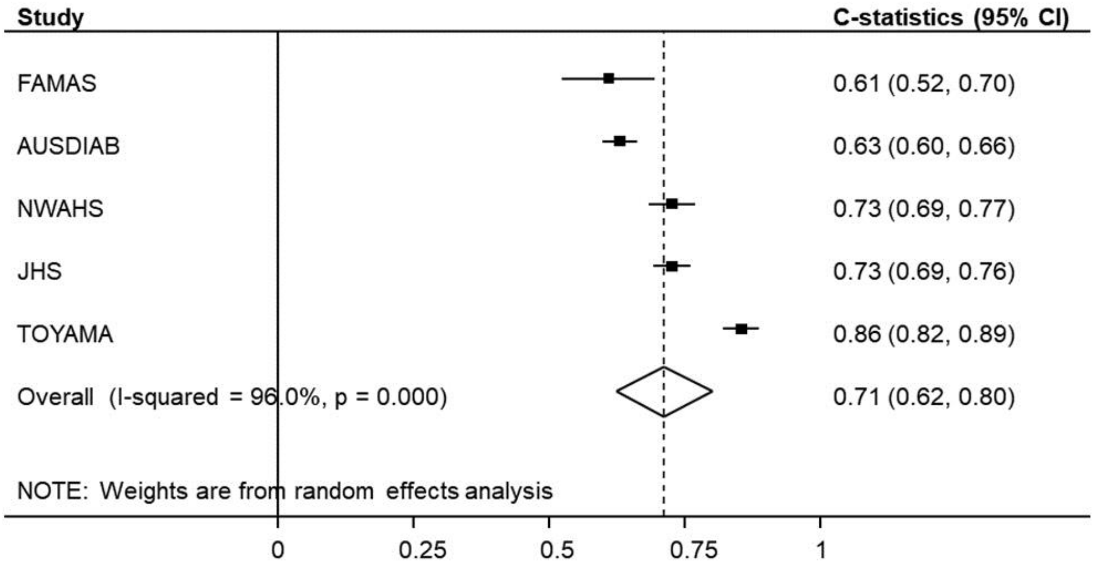

Normal HbA1c (HbA1c<6.0% (42 mmol/mol)) was the reference group;

Lee CMY, *et al. BMJ Open Diab Res Care* 2019; 7:e000794. doi: 10.1136/bmjdrc-2019-000794

sFigure 11: Relationships between each of three measures of glycaemia and incident diabetes

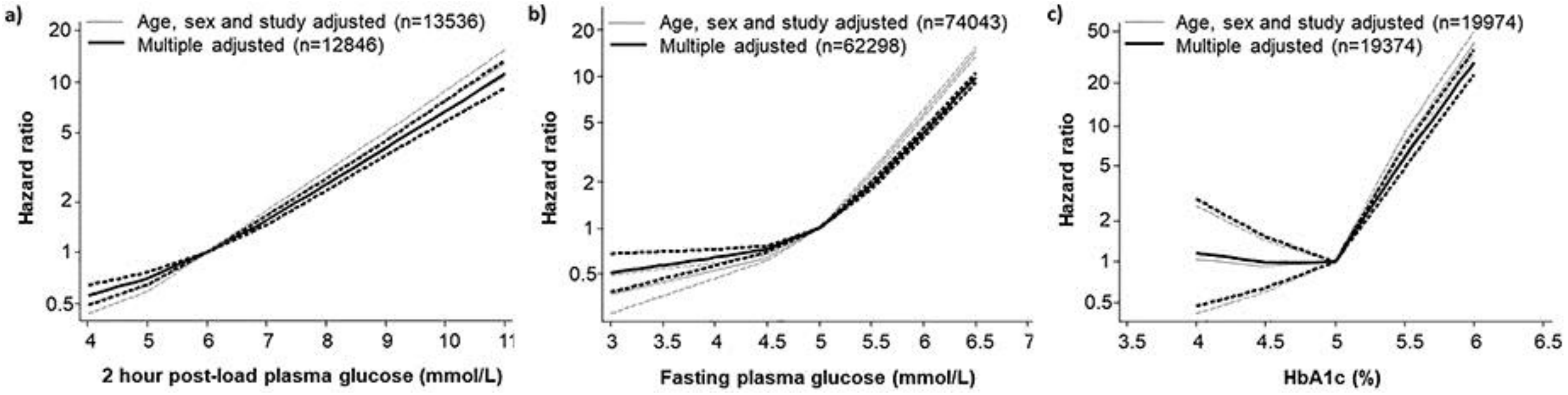

a) Knots at fasting plasma glucose (FPG) 4.2, 4.9, 5.4, and 6.2 mmol/L (76, 88, 97, 112 mg/dl) and reference at 5.0 mmol/L (90 mg/dl); b) knots at 2 hour post-load plasma glucose (2hPG) 3.4, 5.1, 6.2, and 8.9 mmol/L (61, 92, 112, 160 mg/dl) and reference at 6.0 mmol/L (108 mg/dl); c) knots at HbA1c 4.7, 5.1, 5.4, and 6.0% and reference at 5.0%;

Multiple adjusted = adjusted for age, sex, study, body mass index, systolic blood pressure, smoking, and family history of diabetes;

Lee CMY, *et al. BMJ Open Diab Res Care* 2019; 7:e000794. doi: 10.1136/bmjdrc-2019-000794
